# Cognitive load, prior knowledge, and sustained learning intention in a generative-AI-supported digital cultural learning context

**DOI:** 10.3389/fpsyg.2026.1897481

**Published:** 2026-07-03

**Authors:** Hongqing Huang, Qiongxue Zhao, Buling Xia

**Affiliations:** 1 School of Innovation, Hubei Institute of Fine Arts, Wuhan, China; 2 School of Humanities and Law, Northeastern University, Shenyang, China; 3 Department of Visual Design, Graduate School, Hanyang University, Seoul, Republic of Korea

**Keywords:** cognitive load, digital cultural learning, educational psychology, generative AI-supported learning, perceived interactivity, prior knowledge, sustained learning intention

## Abstract

**Background:**

Generative artificial intelligence is increasingly embedded in learning as a source of explanation, feedback, and interactive support. However, it remains unclear how such support shapes learners' cognitive processing and their subsequent willingness to continue learning.

**Objectives:**

From an educational psychology perspective, this study examines how information quality, perceived ease of use, and perceived interactivity are associated with sustained learning intention through intrinsic and extraneous cognitive load, and whether prior cultural knowledge moderates the associations between cognitive load and sustained learning intention.

**Methods:**

This cross-sectional survey study was conducted in The Art of Life: Mawangdui Han Culture Immersive Digital Exhibition as a high-complexity digital cultural learning context. Survey data were collected from 572 learners who reported a recent and complete experience of using generative AI tools for understanding or further learning content related to Mawangdui Han culture. Partial least squares structural equation modeling was used to test net effects, statistical indirect associations, and moderation, and fuzzy-set qualitative comparative analysis was used to identify configurations associated with high and low sustained learning intention.

**Results and conclusions:**

Information quality and perceived ease of use were significantly negatively associated with both intrinsic and extraneous cognitive load, whereas perceived interactivity was significantly positively associated with both forms of load. Intrinsic and extraneous cognitive load were both negatively associated with sustained learning intention. Prior cultural knowledge weakened the negative association between extraneous cognitive load and sustained learning intention but strengthened the negative association between intrinsic cognitive load and sustained learning intention. The fsQCA results further revealed multiple asymmetric configurations associated with high and low sustained learning intention. These findings suggest that the value of generative AI-supported learning in this context lies not in maximizing interaction, but in providing cognitively manageable support. In this study, sustained learning intention refers to a self-reported intention measure rather than observed long-term learning behavior.

## 1 Introduction

Generative artificial intelligence is increasingly embedded in learning as a source of explanation, feedback, and interactive support. This development is especially salient in high-complexity learning contexts, where learners must process unfamiliar concepts, symbolic meanings, historical narratives, and multimodal resources. In China, the Digital Mawangdui initiative and The Art of Life: Mawangdui Han Culture Immersive Digital Exhibition provide a representative setting in which learners use generative AI, digital humans, and intelligent question-answering systems to obtain explanations and feedback during cultural learning (Miao and Holmes, 2021; Miao et al., 2024). However, it remains unclear whether such support reduces learners' cognitive burden or creates additional processing demands. In this study, generative AI-supported learning refers to the process through which learners obtain explanations, feedback, and extension support from generative AI in a digital learning environment, whereas sustained learning intention refers to learners' continuing tendency to remain engaged in related learning activities after the experience (Tang et al., 2023).

As digital cultural learning has shifted from the digital presentation of cultural resources to the question of how learning processes are genuinely supported by digital and AI technologies, existing research has developed along three major paths. The first path is grounded in technology acceptance and post-adoption perspectives. It treats the continued use of digital learning systems or generative AI tools as a behavioral outcome based on cognitive evaluation and focuses on information quality, system quality, perceived usefulness, perceived ease of use, satisfaction, confirmation, and perceived value. These studies typically follow the logic of technological and informational features, individual evaluation, and continuance intention. They show that continued use depends not only on initial acceptance but also on subsequent judgments such as satisfaction, perceived usefulness, and quality perceptions. However, perceived ease of use, flow experience, perceived value, privacy risk, and problematic use may also reshape this mechanism, indicating that continuance behavior cannot be fully explained by general technology acceptance logic (Roca et al., 2006; Zhou and Ma, 2025; Moghavvemi and Jam, 2025; Yan et al., 2024; Susanto et al., 2025).

The second stream emphasizes cognitive and emotional mechanisms in learning. It examines how generative AI, mobile intelligent support, AR, VR, and digital games reshape the distribution of cognitive load, anxiety, cognitive engagement, and learning performance. This line of work does not regard technology as a merely usable tool; rather, it conceptualizes technology as a process factor that reorganizes task difficulty, feedback modes, and cognitive processing paths. Prior studies generally show that scaffolding, immediate feedback, layered annotation, narrative prompts, and moderate interaction can reduce extraneous cognitive load, enhance germane processing, and improve engagement and performance. At the same time, multimodal accumulation, excessive interaction, and inappropriate task design may increase cognitive load and weaken understanding (Sharma and Khadka, 2026; Zhou et al., 2025; Yilmaz et al., 2025; He and Wu, 2023; Li et al., 2026).

The third stream focuses on digital learning contexts of cultural heritage, including virtual museums, digital exhibitions, digital-human guidance, narrative AR, and immersive cultural experiences. It explores how content quality, interactivity, visual aesthetics, emotional involvement, and hedonic experience jointly affect learning motivation, knowledge acquisition, and subsequent participation. A shared understanding in this field is that technology does not operate in isolation in cultural learning. Whether learners are willing to continue engaging often depends on whether they can form cognitive engagement, emotional connection, and a sense of presence in a specific context (Li et al., 2025; Shi et al., 2025a; Mo et al., 2026; Hao et al., 2025).

Overall, existing studies provide important foundations from the perspectives of technology acceptance, cognitive processing, and situated experience, yet their judgments about core drivers remain inconsistent. This divergence reveals at least two gaps. First, technology acceptance, cognitive load, and subsequent learning outcomes are often discussed separately, and a systematic mechanism integrating generative AI support, cognitive processing, and sustained learning outcomes remains underdeveloped. Prior research explains related phenomena through separate routes: technology acceptance studies explain why systems are adopted or continuously used; cognitive load studies examine task processing and learning performance; and cultural heritage learning studies emphasize situated experience and participation. How generative AI affects learners' subsequent learning tendencies through cognitive processing remains insufficiently integrated (Roca et al., 2006; Sharma and Khadka, 2026; Li et al., 2025). Second, existing studies focus more on adoption intention, continuance intention, satisfaction, or short-term learning effects, while paying less attention to a more educationally meaningful outcome: whether learners are willing to deepen related cultural learning after a digital experience. In other words, willingness to use a technology does not equal willingness to continue learning, and continued system use does not necessarily mean that cultural learning continues (Zhou and Ma, 2025; Moghavvemi and Jam, 2025; Susanto et al., 2025).

These gaps are particularly salient in the present context. Digital cultural learning scenarios represented by Mawangdui digital exhibitions are characterized by high information density, multilayered knowledge, long interpretive chains, and the accumulation of multimodal cues. Learners must not only receive information but also select, integrate, and construct meaning across historical contexts and cultural symbols. Prior studies suggest that content complexity and information richness in cultural heritage learning may trigger cognitive overload, learning fatigue, and reduced efficiency. Whether a digital cultural experience can be transformed into deeper cultural engagement also depends on psychological transformation through perceived cultural value and cultural identity, rather than on technological presentation or short-term interaction alone (Zhou et al., 2025; Meng and Dolah, 2025). In this process, generative AI is no longer limited to information supplementation; it further intervenes in content organization, explanatory sequence, and understanding pathways. Ignoring cognitive load may overestimate the positive effects of technological enhancement, whereas ignoring sustained learning intention may mistake one-off interest or short-term interaction for genuine sustained learning engagement.

Accordingly, this study examines learners' perceptions in a generative-AI-supported digital cultural learning context and constructs an explanatory framework linking external support cues, cognitive load, prior knowledge, and sustained learning intention. The novelty of the study lies in three aspects. First, it distinguishes continued learning from continued system use and focuses on sustained learning intention as a more educationally meaningful outcome. Second, it explains this outcome through a psychological mediation mechanism centered on intrinsic and extraneous cognitive load. Third, it combines net-effect analysis (PLS-SEM) and configurational analysis (fsQCA) to reveal both dominant associations and heterogeneous pathways. Three research questions are proposed:

RQ1: In generative AI-supported digital cultural learning, how are external support cues, including information quality, perceived ease of use, and perceived interactivity, associated with learners' intrinsic and extraneous cognitive load?

RQ2: How are different dimensions of cognitive load associated with learners' sustained learning intention, and does prior cultural knowledge moderate these associations?

RQ3: Do the condition combinations leading to high sustained learning intention exhibit multiple pathways, and do their formation logics differ from those leading to low sustained learning intention?

To answer these questions, this study adopts S-O-R as the overarching theoretical framework. Information quality, perceived ease of use, and perceived interactivity are defined as stimuli; intrinsic cognitive load and extraneous cognitive load are defined as organism variables; and sustained learning intention is defined as the response outcome. Methodologically, a two-step analytical strategy is adopted. First, partial least squares structural equation modeling (PLS-SEM) is used to test net associations, statistical indirect associations, and moderation. Second, fuzzy-set qualitative comparative analysis (fsQCA) is used to identify multiple configurations leading to high and low sustained learning intention (Xia et al., 2026; Zheng et al., 2023). The theoretical contribution of this study lies in explaining sustained learning as a cognitively mediated outcome rather than as a direct extension of technology adoption. Methodologically, the study combines net-effect analysis and configurational analysis to reveal both dominant mechanisms and heterogeneous pathways. Practically, it provides evidence for the design of AI-supported learning environments in high-complexity knowledge contexts.

## 2 Literature review, theoretical framework, and hypothesis model

### 2.1 Literature review and current research

#### 2.1.1 AI-supported digital cultural learning and subsequent engagement

Existing research on how AI supports digital cultural learning has formed a research lineage centered on virtual museums, digital heritage displays, cultural heritage serious games, and immersive guidance. Its primary concern is whether digital and intelligent technologies can improve the presentation and accessibility of traditional cultural content, thereby enhancing learners' identification, participation, and subsequent action. In a virtual museum study, Meng et al. used SEM based on SOR and the information systems success model and found that external stimuli such as information quality and perceived interactivity promote offline visit intention through perceived cultural value and cultural identity. This indicates that research on digital cultural learning has begun to move from platform experience toward sustained cultural participation (Meng and Dolah, 2025). Iddamalgoda et al. employed questionnaires and semi-structured interviews in an interactive cultural heritage exhibition and, based on AI-TAM, found that trust significantly enhances perceived usefulness and perceived ease of use and influences behavioral intention through attitude. This suggests that generative AI has been regarded as an effective tool for supporting cultural learning and reflection rather than a general information-retrieval technology (Iddamalgoda et al., 2026).

In terms of outcome variables, this field has gradually moved beyond the early focus on whether technology is accepted and has turned toward more educationally meaningful subsequent outcomes. Studies have examined adoption intention, satisfaction, continuance intention, cultural identity, and sustainable heritage engagement. A relatively stable view has emerged: AI and digital technologies can enhance interactivity and immersion in digital cultural learning, but their value lies not merely in stimulating short-term interest, but in whether they foster longer-term cultural understanding, identification, and engagement (Meng and Dolah, 2025). Nevertheless, the boundary of this research stream is also clear. Existing outcomes often remain at the level of platform continuance or offline visit intention, while relatively few studies directly examine whether learners are willing to continue encountering, understanding, and investing in digital cultural learning as a core outcome. This creates room for the present study to focus on sustained learning intention (Meng and Dolah, 2025).

#### 2.1.2 External support cues, cognitive processing, and post-learning engagement

A second body of research directly related to the present model centers on the chain from external technological features to internal processing mechanisms and sustained learning outcomes. In terms of antecedents, information quality, perceived ease of use, interactivity, system quality, personalization, and learning community are among the most common explanatory starting points. Zheng et al. found in e-learning research that perceived ease of use and perceived usefulness significantly affect intention to continue e-learning, while external quality factors such as system quality, personalization, and learning community operate indirectly through internal perceptual variables. This suggests that sustained learning outcomes are usually not driven by a single factor but jointly shaped by external stimuli and internal evaluations (Zheng et al., 2023). Similarly, Alwakid et al. used SEM based on SOR in AI chatbot-supported learning and found that PEOU, PEU, ANM, and PIQ influence intention to use through satisfaction and trust, situating the discussion within sustainable learning and showing that research on learning support technologies is gradually moving from adoption toward long-term educational value (Alwakid et al., 2025).

If these studies answer which technological and informational features promote long-term learning outcomes, cognitive load research further explains how this influence occurs during learning processing. Zhou et al. proposed a three-layer interactive annotation model in a cultural heritage serious game based on cognitive load theory and showed through quantitative and qualitative analyses that when information presentation and task complexity are layered and dynamically adjusted, learners' extraneous load decreases and germane load is optimized, thereby improving cultural knowledge understanding, retention, and emotional engagement (Zhou et al., 2025). Likewise, Li et al., in a serious game study on traditional Chinese murals based on scaffolding theory, found through experimental comparison that appropriate supportive design not only enhances learning interest but also helps learners better understand cultural connotations (Li et al., 2024). This indicates that, in contexts where cultural content is abstract and representationally complex, learning outcomes depend strongly on whether cognitive processing is adequately supported (Li et al., 2024).

However, existing findings are not entirely consistent on key paths. Most studies suggest that information quality, perceived ease of use, and interactivity steadily promote continued learning, but others indicate that the effects of technological support variables are not always linearly positive (Almahamid et al., 2010; Gupta et al., 2021). Buling Xia et al. found in a study of text-to-image educational use that perceived ease of use and information quality do not necessarily reduce anxiety, and may coexist with trust amid anxiety. This indicates that the effects of technological features are shaped by task uncertainty, risk perception, and contextual complexity (Xia et al., 2026). This suggests that if research only discusses whether technological features are useful without incorporating process variables such as cognitive load, it is difficult to explain why learners move from initial exposure to sustained learning intention.

Prior studies also indicate the boundary role of prior knowledge. Sáiz Manzanares et al. used eye-tracking, ANCOVA, and data-mining techniques in an art history learning task to compare learners with and without prior knowledge. They found that learners with prior knowledge demonstrated more effective cognitive strategies and better learning outcomes, suggesting that prior knowledge affects how learners process complex cultural information and how efficiently they do so (Sáiz Manzanares et al., 2020). This provides direct inspiration for examining the moderating role of prior cultural knowledge in generative AI-supported digital cultural learning.

#### 2.1.3 Specific context, theoretical integration, and methodological boundaries

Although prior research provides important foundations regarding AI applications, sustained learning outcomes, and cognitive load mechanisms, three limitations remain with respect to generative AI-supported digital cultural learning (Dong et al., 2025). First, contextual embedding is insufficient. Existing studies mostly derive from general e-learning, virtual museums, AI chatbots, or digital heritage platforms and focus on adoption, continuance, satisfaction, or offline visit intention, while paying less attention to how digital cultural learning continues (Pegolo, 2023; Bakhnoo et al., 2026). Second, theoretical integration is insufficient. Technology acceptance research usually employs TAM, UTAUT, D&M, or SOR to explain how external stimuli affect adoption and continuance, whereas cognitive load research is usually grounded in CLT to explain how task design and information granularity influence understanding and retention. The former rarely addresses cognitive burden in complex cultural learning, while the latter rarely incorporates cognitive load and sustained learning intention into one explanatory chain. A unified framework linking external technological support features, internal cognitive load mechanisms, and sustained learning intention remains lacking (Zhou et al., 2025; Zheng et al., 2023). Third, methodological boundaries remain evident. Existing studies rely mainly on single linear analyses and emphasize average net effects among variables, while rarely revealing how different condition combinations jointly produce sustained learning outcomes. Prior research shows that high intention to continue e-learning is not caused by a single service-quality factor but by multiple condition combinations; high sustainable use intention and low intention also exhibit clear configurational asymmetry, indicating that subsequent outcomes in complex technology-based learning contexts are heterogeneous and configurational (Xia et al., 2026; Zheng et al., 2023). SEM-type linear methods can therefore identify average paths but struggle to explain why different learners form similar or opposite outcomes through different condition combinations (Machlanski et al., 2024).

In sum, existing studies provide three foundations for this study. First, AI involvement in digital cultural learning has become a clear trend, and related research has moved from technological presentation toward cultural value, cultural identity, and long-term participation. Second, information quality, perceived ease of use, interactivity, cognitive load, and prior knowledge have been shown in adjacent fields to be closely related to long-term learning outcomes. Third, theories such as SOR, TAM, D&M, and CLT provide useful foundations for explaining the relationship among external stimuli, internal processing, and subsequent behavior (Zhou et al., 2025; Meng and Dolah, 2025; Zheng et al., 2023). Nevertheless, an integrated explanation of the specific context of generative AI-supported digital cultural learning remains lacking, especially one that unifies AI support features, cognitive load, and sustained learning intention while revealing both net effects and configurational mechanisms.

### 2.2 Embedded theoretical foundations

#### 2.2.1 Stimulus-organism-response framework

The S-O-R framework holds that individual behavior is not directly determined by the external environment but is formed through the mediating role of internal cognitive and affective states (Zhu et al., 2020). It has been widely used to explain digital learning, continued use of online platforms, virtual museum experience, and cultural participation because it effectively integrates the chain of technological features, psychological processing, and behavioral outcomes (Meng and Dolah, 2025; Zheng et al., 2023). Some studies have treated virtual exhibition experience quality as the stimulus, perceived cultural value and cultural identity as organism states, and offline visit intention as the behavioral response, thereby explaining how digital cultural experience is transformed into subsequent cultural participation (Meng and Dolah, 2025). In this study, S-O-R functions as the overarching framework. Information quality, perceived ease of use, and perceived interactivity constitute the stimulus layer; intrinsic cognitive load and extraneous cognitive load constitute the organism layer, reflecting learners' internal cognitive states when understanding, selecting, and processing traditional cultural content; and sustained learning intention constitutes the response layer, reflecting whether learners are willing to continue investing in related learning after an experience. This mapping conforms to the basic logic of S-O-R and fits the AI support-cognitive processing-sustained learning intention chain examined in this study (Li et al., 2025; Meng and Dolah, 2025).

#### 2.2.2 Cognitive load theory

Cognitive load theory emphasizes that learning outcomes depend on how limited working-memory resources are allocated during learning tasks. Intrinsic cognitive load is mainly related to element interactivity, task complexity, and learners' prior knowledge, whereas extraneous cognitive load is generated by ineffective information presentation, interface organization, or instructional design (Sweller, 2010). The complexity inherent in the learning task generates intrinsic cognitive load, whereas inappropriate information presentation, interface organization, and instructional design increase extraneous cognitive load. In cultural heritage learning, serious games, and immersive learning research, CLT has been used to explain why complex cultural content easily leads to understanding difficulties and which supportive designs can improve knowledge acquisition and retention (Zhou et al., 2025; Li et al., 2024). Prior studies have shown that layered tasks, interactive annotations, and scaffolding support can reduce extraneous cognitive load and promote more effective cultural knowledge processing (Zhou et al., 2025; Li et al., 2024). In this study, CLT is introduced to define the core mechanism of the organism layer. In generative AI-supported digital cultural learning, traditional cultural content is often symbolically dense, semantically abstract, and characterized by long interpretive chains. Learners must process not only the complexity of cultural content itself but also multimodal information, interface operations, and prompt feedback provided by AI. Therefore, intrinsic cognitive load is interpreted as learners' perceived intrinsic cognitive burden when processing historically dense, semantically interdependent, and conceptually layered cultural content in an AI-supported environment, rather than as a purely objective indicator of task-inherent load. Extraneous cognitive load refers to additional perceived burden associated with information organization, interface pathways, feedback modes, and interaction design (Zhou et al., 2025; Gorbunova et al., 2024). Germane load was not included as a focal construct because the present model was designed to examine how perceived support cues are associated with experienced cognitive burden and sustained learning intention. Future research may incorporate germane load to capture productive processing more explicitly.

#### 2.2.3 Technology acceptance model

The technology acceptance model argues that individuals' intention to use technology depends primarily on their cognitive evaluation of the technology's usability, especially perceived usefulness and perceived ease of use (Marangunić and Granić, 2015). Although TAM was initially developed for information-system adoption, it has been widely applied to e-learning and educational use of generative AI to explain why learners form positive attitudes, continuance intention, or long-term learning engagement (Zheng et al., 2023; Iddamalgoda et al., 2026; Guo et al., 2025). Prior research shows that perceived ease of use affects not only initial acceptance but also long-term learning outcomes through satisfaction, trust, or continuance intention (Zheng et al., 2023; Guo et al., 2025). In this study, TAM is not used to reconstruct a complete technology acceptance chain; rather, it provides the theoretical source for perceived ease of use as a key stimulus variable. In generative AI-supported digital cultural learning, if learners perceive the technology as easy to understand, prompts as easy to master, and interaction as clear and controllable, they are more likely to form positive technology judgments and maintain subsequent learning engagement. TAM therefore supplements the stimulus layer by explaining how technological cues enter learners' internal processing and become connected to sustained learning intention (Iddamalgoda et al., 2026; Guo et al., 2025).

#### 2.2.4 Theoretical integration

The theoretical integration in this study is not a simple juxtaposition of several theories but a layered embedding based on the functional position of each variable in the model. S-O-R is used as the overarching analytical framework because generative AI-supported digital cultural learning is essentially a continuous process triggered by external support cues, mediated by internal cognitive processing, and ultimately transformed into subsequent learning responses (Li et al., 2025; Zheng et al., 2023). Accordingly, information quality, perceived ease of use, and perceived interactivity are placed in the stimulus layer. Information quality represents content support cues; perceived interactivity represents interaction support cues; and perceived ease of use is theoretically grounded in TAM and represents technological usability cues (Iddamalgoda et al., 2026; Guo et al., 2025). Intrinsic and extraneous cognitive load are placed in the organism layer and explained through CLT because they represent neither external technology features nor final learning outcomes, but learners' internal cognitive states after receiving external support. Intrinsic cognitive load reflects the necessary processing demands caused by the complexity of traditional cultural content, whereas extraneous cognitive load reflects the additional cognitive burden caused by information organization, feedback modes, and interaction pathways (Zhou et al., 2025; Gorbunova et al., 2024). Sustained learning intention is placed in the response layer because it reflects whether learners are willing to continue encountering, understanding, and investing in related digital cultural learning after such cognitive processing (Guo et al., 2025; Fu et al., 2020). Prior cultural knowledge is not placed in the main S-O-R chain but is treated as a boundary condition that moderates the effect of cognitive load on sustained learning intention because existing schemas and knowledge reserves influence learners' processing efficiency and allocation of cognitive resources when dealing with complex cultural information (Cai et al., 2025; Gorbunova et al., 2025). Therefore, this study uses S-O-R to organize the overall hierarchy, TAM to explain perceived ease of use in the stimulus layer, CLT to explain cognitive load in the organism layer, and prior cultural knowledge as a boundary condition outside the main chain, forming an integrated framework of external support cues, cognitive load, and sustained learning intention.

### 2.3 Hypothesis model

#### 2.3.1 Information quality (IQ)

Information quality is a classic construct in information systems research and generally refers to the overall quality of system output in terms of accuracy, completeness, relevance, timeliness, and understandability (Al-Mamary et al., 2014). In digital museum and generative AI education research, Shi et al. and Xia et al. further define it as users' overall judgment of the content itself and its presentation, commonly measured through accuracy, completeness, relevance, timeliness, and format appropriateness (Xia et al., 2026; Shi et al., 2025b). In this study, information quality refers to learners' overall perception of the accuracy, relevance, completeness, clarity, and understandability of digital cultural learning information provided by generative AI. Based on S-O-R and CLT, high-quality information can reduce unnecessary processing caused by ambiguity, redundancy, and irrelevant cues, thereby lowering ineffective cognitive expenditure during information search, evaluation, and integration and reducing extraneous cognitive load. At the same time, clearly structured and relevant information may make complex cultural content feel more manageable and may therefore be associated with lower perceived intrinsic cognitive burden during processing. Zheng et al. identified information quality as an important external stimulus associated with learners' internal cognitive evaluation and sustained learning outcomes in e-learning (Zheng et al., 2023), and Zhou et al. showed that optimized information granularity and layered annotations can reduce extraneous cognitive load and improve knowledge understanding and retention in cultural heritage serious games (Zhou et al., 2025). Therefore, H1a: Information quality has a significant negative association with intrinsic cognitive load. H1b: Information quality has a significant negative association with extraneous cognitive load.

#### 2.3.2 Perceived ease of use (PEOU)

Perceived ease of use is a core construct in TAM and refers to the degree to which an individual believes that using a system requires little effort (Davis, 1989). In educational technology and digital culture learning, PEOU usually describes learners' overall perception of whether the interface, functional paths, interaction logic, and task operations are clear, convenient, and easy to master (Yan et al., 2024; Guo et al., 2025). In this study, PEOU refers to learners' overall perception of whether system operation, prompt input, result invocation, and the learning process are clear, simple, and low-effort in generative AI-supported digital cultural learning. Higher PEOU means that learners can complete learning tasks with lower costs of interface understanding, path search, and functional control, reducing extra processing unrelated to learning goals and directly alleviating extraneous cognitive load. When technological interaction is easier to master, learners can allocate more cognitive resources to cultural content and develop stronger task controllability, which may make complex cultural materials feel more manageable and may therefore be associated with lower perceived intrinsic cognitive burden. Prior studies show that PEOU is associated with continuous intention to use and sustained learning intention (Yan et al., 2024; Guo et al., 2025). Lai et al. further noted that TAM alone cannot fully explain behavioral formation in multimodal cultural experiences if cognitive processing is ignored, suggesting a close link between technological operability and cognitive load (Lai and Zhang, 2025). Therefore, H2a: PEOU has a significant negative association with intrinsic cognitive load. H2b: PEOU has a significant negative association with extraneous cognitive load.

#### 2.3.3 Perceived interactivity (PINT)

Perceived interactivity refers to learners' subjective perception of whether a system provides timely, two-way, and controllable interaction (Cheng, 2014). Its core lies not in the number of functions but in whether users feel responded to, understood, and able to guide the interaction process. Lyu et al. conceptualize it as learners' overall experience of control, system responsiveness, and meaningful participation in AI systems (Lyu et al., 2026), whereas Alwakid et al. treat it as a key external stimulus in intelligent learning support contexts (Alwakid et al., 2025). In this study, perceived interactivity refers to learners' overall perception of whether generative AI provides timely feedback, personalized responses, process control, and sustained interaction support during digital cultural learning. High-quality interaction can reduce ineffective searching, repeated trial and error, and interface switching through immediate feedback, dynamic prompts, and learner control, thereby potentially reducing extraneous cognitive load. Personalized explanations and adjustable interaction can also unfold complex cultural content step by step, enhance task controllability, and make perceived task complexity more manageable. Lyu et al. found that personalization, learner control, and engagement in AI interactivity significantly enhance behavioral intention through perceived usefulness and perceived ease of use (Lyu et al., 2026). Meng et al. found that perceived interactivity promotes perceived cultural value and cultural identity in virtual museums (Meng and Dolah, 2025). From an educational psychology perspective, perceived interactivity is expected to be associated with post-learning engagement not directly, but through its influence on learners' processing demands during AI-supported learning. However, in high-complexity learning contexts, interactivity may also introduce coordination, selection, and integration demands, meaning that its relationship with perceived cognitive burden may not always be negative. Wu et al. similarly cautioned that interaction may increase cognitive load if it only adds mechanical operations without effective feedback (Wu et al., 2024). Therefore, H3a: PINT has a significant negative association with intrinsic cognitive load. H3b: PINT has a significant negative association with extraneous cognitive load.

#### 2.3.4 Intrinsic cognitive load (ICL)

Intrinsic cognitive load is a core construct in cognitive load theory and is usually associated with task complexity, element interactivity, and learners' prior knowledge (Kala and Ayas, 2023). It originates primarily from content complexity rather than external presentation. Following this line of research, this study defines ICL as learners' perceived intrinsic cognitive burden when dealing with the historical context, symbolic meanings, and knowledge relationships of traditional cultural content during generative AI-supported digital cultural learning (Gorbunova et al., 2024). Higher ICL means that learners perceive that they must invest substantial cognitive resources in understanding cultural concepts, integrating background information, and constructing meaning relations. When such perceived necessary processing continues to occupy limited resources, learners may become less willing to continue related learning after the focal experience. Zhou et al. noted that the intrinsic richness and complexity of cultural information in cultural heritage serious games can trigger cognitive overload, learning fatigue, and reduced efficiency (Zhou et al., 2025). Qi et al. found that when GenAI optimizes intrinsic load, tasks become more manageable, indirectly suggesting that high ICL may be negatively associated with learning sustainability (Qi et al., 2025). Sharma and Khadka also showed that intrinsic cognitive load remains an important constraint in complex mobile AI learning tasks (Sharma and Khadka, 2026). Therefore, H4: ICL has a significant negative association with sustained learning intention.

#### 2.3.5 Extraneous cognitive load (ECL)

Extraneous cognitive load refers to the additional cognitive burden caused by inappropriate information presentation, interface organization, and instructional design (Skulmowski and Xu, 2022). It does not originate from learning content itself but from the learning environment's ineffective occupation of working memory. In this study, ECL refers to the unnecessary perceived cognitive processing burden associated with unclear information presentation, redundant feedback, complex interaction paths, or fragmented interface operations during generative AI-supported digital cultural learning. Higher ECL occupies working-memory resources needed for understanding cultural content, integrating historical context, and establishing meaning connections, thereby weakening learning fluency, task controllability, and subsequent engagement intention. Prior studies show that ECL can significantly impair learning performance in serious games on traditional Chinese murals (Qi et al., 2025). Sharma and Khadka argued that a key mechanism by which generative AI improves learning experience is clarifying fragmented content, reducing extraneous load, decreasing learning anxiety, and improving continued engagement (Sharma and Khadka, 2026). Qi et al. (2025) similarly noted that complex tasks become more manageable when GenAI reduces additional costs in information integration and processing. Therefore, H5: ECL has a significant negative association with sustained learning intention.

#### 2.3.6 Sustained learning intention (SLI)

Sustained learning intention refers to learners' behavioral tendency to continue participating in, deepening, and maintaining related learning activities after the current learning experience (Ben-Eliyahu, 2021; Shephard, 2008). It distinguishes one-off use from sustained learning engagement. In AI-supported learning, Fu et al. define it as learners' intention to continue subsequent learning activities after using AI learning tools and argue that it more directly reflects the psychological basis of learning persistence and retention (Fu et al., 2020). In AR education, Guo et al. regard it as learners' long-term tendency to continue participating in learning activities with technological support, emphasizing that it differs from general use intention and is closer to long-term learning commitment (Guo et al., 2025). Therefore, this study defines SLI as learners' behavioral tendency to continue encountering, understanding, and investing in related digital cultural learning after a generative AI-assisted learning experience.

#### 2.3.7 Prior cultural knowledge (PCK)

Prior cultural knowledge refers to learners' pre-existing knowledge, experience, and understanding related to the target cultural topic before entering a specific learning context. Cai et al. (2025) note that prior knowledge is the learner's knowledge reserve in a specific domain and affects learning performance and support needs. Gorbunova et al. emphasize that prior knowledge represents existing schemas that learners can mobilize, affecting the difficulty and efficiency of processing new information (Gorbunova et al., 2025). In this study, PCK refers to learners' knowledge reserve related to relevant cultural content, historical background, and core concepts before using generative AI for digital cultural learning. Based on CLT and schema theory, higher PCK helps learners use existing schemas to understand new information and may buffer the inhibiting effect of cognitive load on subsequent learning engagement. Cai et al. found that prior knowledge significantly changes the effects of learning support design on cognitive load and learning outcomes (Cai et al., 2025), while Huangfu et al. found that learners with higher prior knowledge generally show lower intrinsic and extraneous cognitive load (Huangfu et al., 2026). Therefore, H6a: PCK weakens the negative association between ICL and SLI. H6b: PCK weakens the negative association between ECL and SLI.

## 3 Research design and methods

### 3.1 Research context and case selection

This study takes The Art of Life: Mawangdui Han Culture Immersive Digital Exhibition as the focal digital cultural learning context and examines learners' perceived experience of generative AI-supported learning in relation to Mawangdui Han culture. Mawangdui Han culture is characterized by high information density, multilayered historical background, symbolic complexity, and long interpretive chains. Learners are therefore required to select, integrate, and construct meaning from complex cultural information. The exhibition integrates immersive digital displays, images, videos, spatial narrative, and interactive presentation, making it a suitable high-complexity learning context for examining perceived cognitive load in AI-supported cultural learning.

In this study, “generative AI-supported learning” refers to learners' self-reported use of generative AI tools to obtain explanations, feedback, clarification, or extended support while understanding content related to Mawangdui Han culture. This AI-supported experience could involve text-based, multimodal, digital-human, or intelligent question-answering interaction, including public generative AI tools and AI-supported interpretive services associated with the exhibition context. Participants were not required to use one standardized AI platform. Instead, the study captured learners' perceived experiences in a naturally occurring generative-AI-supported digital cultural learning context. Their reported experience could occur during engagement with exhibition-related digital materials or shortly after the visit when using AI tools for further understanding and interpretation.

Accordingly, the present study does not investigate a single experimentally controlled AI intervention, but a survey-based learning context in which learners used generative AI to support cultural understanding. This context is appropriate for examining how perceived external support cues are associated with cognitive load and sustained learning intention in digital cultural learning. Because the study was designed as a perception-based cross-sectional survey rather than an experimental intervention, the duration of AI use, specific prompts, and task sequences were not standardized by the researchers. Respondents were instructed to answer based on their most recent and most complete AI-supported cultural learning experience, and the AI exposure was self-reported rather than directly observed.

It should also be noted that the construct of generative-AI-supported learning in this study refers to learners' retrospective perceptions of heterogeneous AI-supported learning experiences rather than to a single, standardized AI intervention. Participants may have used different AI systems, interaction formats, task purposes, and engagement durations. Therefore, the findings should be interpreted as associations among perceived AI support cues, perceived cognitive load, and sustained learning intention within naturally occurring and heterogeneous AI-supported cultural learning experiences, rather than as evidence about the effects of a clearly specified AI learning environment in general.

### 3.2 Scale design

The model includes three antecedent variables, two mechanism variables, sustained learning intention as the outcome variable, and prior cultural knowledge as a moderator. All variables were measured on a seven-point Likert scale (1 = strongly disagree, 7 = strongly agree). The measurement items were adapted from established scales reported in prior studies (see Appendix A), and the wording was contextually adjusted to fit generative-AI-supported digital cultural learning while preserving the original construct meanings. A summary of the scale sources and adaptation logic is provided in Appendix A. Prior cultural knowledge was measured retrospectively as learners' self-reported pre-existing knowledge base prior to the focal generative-AI-supported learning experience.

### 3.3 Sample and data collection

Data were collected through a cross-sectional questionnaire survey. Eligible respondents were adult learners who had used at least one generative AI tool or AI-supported interpretive service to understand, interpret, or further learn content related to Mawangdui Han culture or exhibition-related digital cultural materials. Respondents were asked to answer based on their most recent and most complete generative-AI-supported cultural learning experience in order to improve response consistency and accuracy. The AI-supported learning experience was self-reported rather than experimentally observed or standardized by the researchers. Because this study was designed as a perception-based survey rather than an experimental intervention, the duration of AI use, specific prompts, and task sequences were not standardized by the researchers.

Respondents were recruited through online questionnaire distribution and relevant learning or exhibition-related networks. The survey was administered anonymously, and participation was voluntary. To ensure eligibility, the questionnaire included experience-authenticity screening items requiring respondents to confirm that they had relevant generative-AI-supported cultural learning experience. Because relevant generative-AI-supported cultural learning experience was used as an inclusion criterion, all valid respondents reported such experience. A total of 665 questionnaires were collected. During data cleaning, responses that failed the experience-authenticity screening or attention-check items, showed abnormal completion times, or contained obvious logical inconsistencies were removed. After this process, 572 valid responses remained, yielding an effective response rate of 86.02%. The excluded responses were treated as a combined invalid-response category because multiple exclusion criteria were applied jointly during data cleaning.

The characteristics of the final valid sample are reported in Table 1. The sample mainly consisted of adult learners with prior museum or digital exhibition experience, and students accounted for a relatively high proportion of the respondents. Therefore, the findings should be interpreted as reflecting perceptions among learners with relevant digital cultural learning experience rather than the general public.

**Table 1:** Sample characteristics and prior experience distribution.

Characteristic	Category	n	%
Gender	Male	307	53.67
	Female	265	46.33
Age	18–20	96	16.78
	21–25	206	36.01
	26–30	175	30.59
	Above 30	95	16.61
Educational level	Undergraduate	263	45.98
	Master's student	238	41.61
	Doctoral student	47	8.22
	Other	24	4.20
Learner status	Student	387	67.66
	Exhibition visitor	75	13.11
	General online respondent with relevant experience	110	19.23
Prior museum/digital exhibition experience	Yes	489	85.49
	No	83	14.51
Prior cultural knowledge level	Low PCK (1.00–3.00)	102	17.83
	Medium PCK (3.01–5.00)	67	11.71
	High PCK (5.01–7.00)	403	70.45

Percentages are calculated based on the final valid sample of 572 respondents. PCK = prior cultural knowledge. PCK level was calculated using the mean score of PCK1–PCK4 on a seven-point Likert scale. This grouping was used only for descriptive reporting and was not used to estimate the moderation model. Prior museum / digital exhibition experience was a self-reported background variable and should not be equated with the PCK construct in the structural model.

This study followed the principles of voluntary participation, anonymous completion, and minimal risk. The study was reviewed and approved by the Ethics Review Committee of the School of Design, Hanyang University, Republic of Korea (approval date: 3 February 2026). Before completing the questionnaire, all respondents were presented with an informed-consent statement and could proceed only after indicating their agreement electronically. Only adult participants aged 18 or above were eligible to participate. No directly identifiable personal information was collected, and all data were processed in de-identified form and reported only in aggregate for academic research purposes.

### 3.4 Data analysis methods

**Figure 1:**
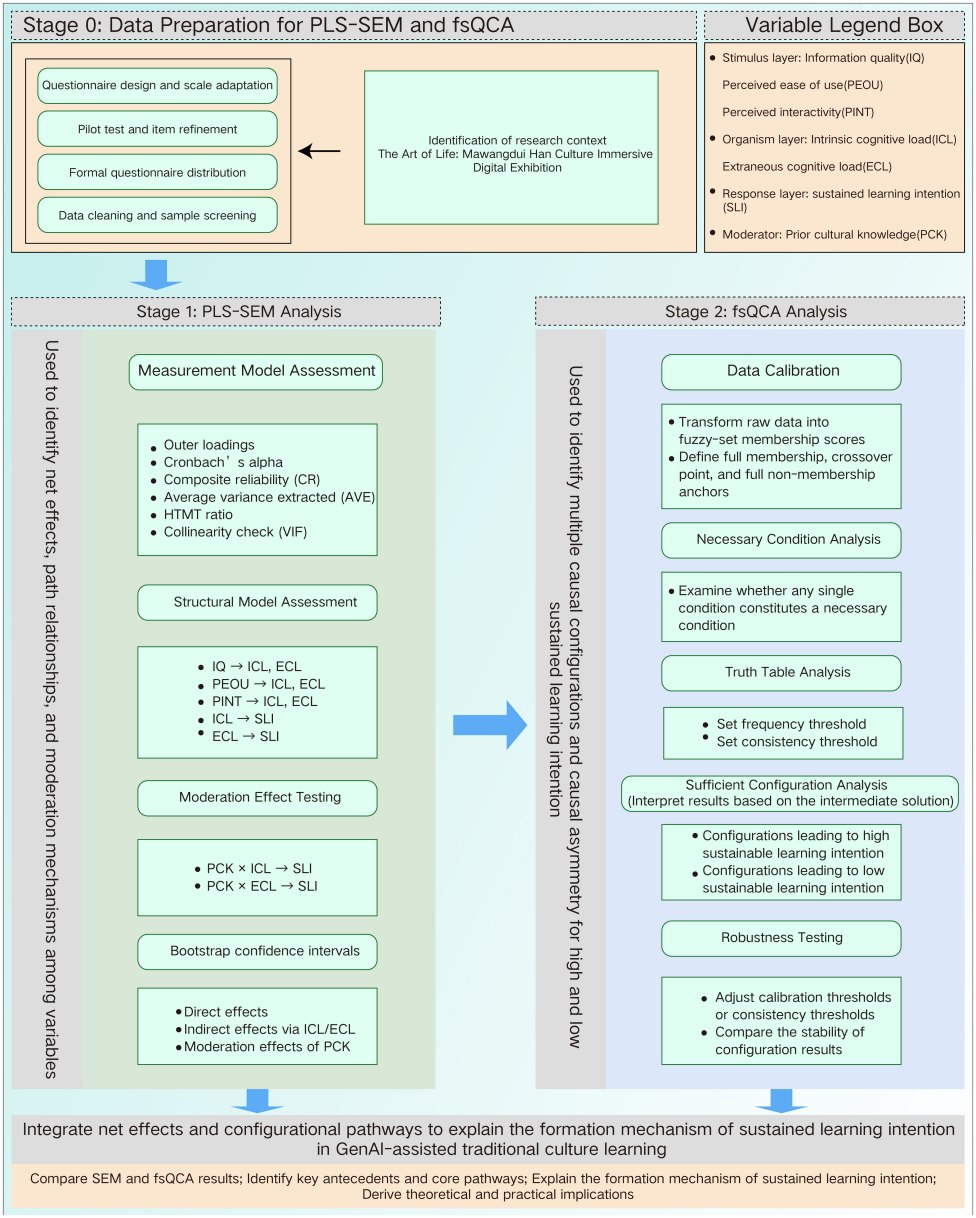
Integrated analytical framework for sustained learning intention based on PLS-SEM and fsQCA.

**Figure 2:**
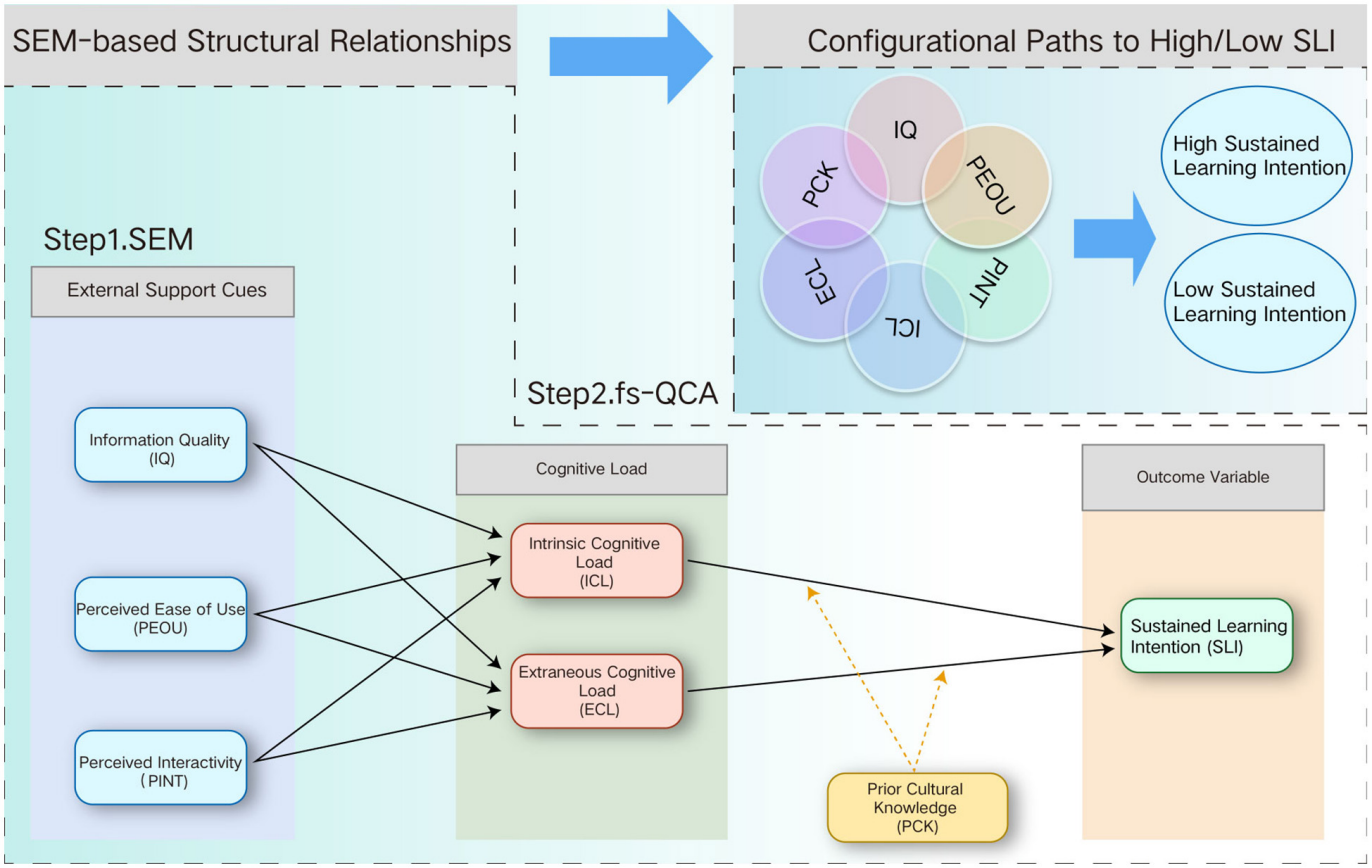
Research model and configurational analysis framework.

This study combines PLS-SEM and fsQCA to examine the theoretical model and its mechanisms. The analysis proceeded in two steps. Figure 1 summarizes the overall analytical procedure, and Figure 2 presents the research model and configurational analysis framework. First, PLS-SEM was used to test linear net associations, path relationships, statistical indirect associations, and moderation (Fassott et al., 2016). All constructs in this study were specified as reflective. PLS-SEM was selected because the model includes multiple latent constructs, indirect paths, and interaction effects, and because the study aims to integrate net-effect analysis with configurational analysis within the same analytical framework. Model estimation was conducted in SmartPLS 4.1, and 5,000 bootstrap resamples were used to test the significance of path coefficients and indirect associations. Invalid responses were removed during data cleaning prior to model estimation. Because the study is based on a cross-sectional self-report design, the PLS-SEM results should be interpreted primarily as explanatory associations rather than strong causal effects. Second, fsQCA was used to identify multiple condition configurations associated with high and low sustained learning intention (Grunwald et al., 2025). Information quality, perceived ease of use, perceived interactivity, intrinsic cognitive load, extraneous cognitive load, and prior cultural knowledge were treated as condition variables, while sustained learning intention was treated as the outcome variable. Following Ragin's direct calibration method (Ragin, 2009), raw data were transformed into fuzzy-set membership scores. The detailed calibration anchors are reported in Appendix B. The truth-table construction settings, including the case-frequency threshold, raw consistency threshold, PRI consistency threshold, and contradictory-configuration handling rule, are reported in Appendix C. The complete truth tables and additional fsQCA solution outputs are reported in Appendices F, G. Robustness-check settings and results are reported in Appendix H. Necessary-condition and sufficient-condition analyses were then conducted, with the intermediate solution used as the primary interpretive basis. Robustness checks were performed by adjusting calibration thresholds or consistency standards. Overall, the combination of PLS-SEM and fsQCA is not a simple methodological addition but is grounded in the configurational complexity of the research object. PLS-SEM identifies net effects and paths among variables, revealing which factors are more important on average and through what mechanisms they operate. fsQCA is better suited to examining whether equifinal pathways exist in generative AI-supported digital cultural learning—that is, whether learners with different technology perceptions, cognitive load states, and cultural knowledge bases form similarly high sustained learning intention through different configurations. Combining the two methods thus reveals both the dominant mechanisms and configurational features of sustained learning intention formation.

## 4 Data analysis results

### 4.1 PLS-SEM results

PLS-SEM was used to examine the structural relationships among external support cues, cognitive load, prior knowledge, and sustained learning intention. The method was chosen because the model includes multiple reflective latent constructs, indirect paths, and moderation effects, and because the study combines net-effect analysis with configurational analysis. Model estimation was conducted in SmartPLS 4.1 with 5,000 bootstrap resamples.

#### 4.1.1 Common method bias test

Because the data were collected mainly through a single questionnaire at one time point, both procedural and statistical remedies were used to assess common method bias. Procedurally, the survey emphasized anonymity, voluntary participation, and the absence of right or wrong answers. Experience-authenticity screening items and attention-check items were included, and invalid responses were removed during data cleaning. Statistically, Harman's single-factor test was first conducted using unrotated principal component analysis for all measurement items. The results extracted seven factors with eigenvalues greater than 1, and the first common factor explained 36.298% of the variance, which was below the commonly used 50% threshold. In addition, full-collinearity VIF values were examined as a supplementary diagnostic. The full-collinearity VIF values ranged from 1.326 to 1.812, below the strict threshold of 3.3, suggesting that common method bias was unlikely to severely distort the results. Nevertheless, because all main constructs were self-reported at one time point, residual common method variance cannot be fully ruled out. The full-collinearity VIF results are reported in Appendix E.

#### 4.1.2 Measurement model evaluation

Before testing the structural model, the measurement model was evaluated. As shown in Table 2, all item loadings exceeded 0.70, Cronbach's alpha and composite reliability were above 0.70, and AVE values were above 0.50, indicating satisfactory internal consistency and convergent validity. The VIF values for all items were below 5, indicating no serious multicollinearity. As shown in Tables 3a, b, under the Fornell-Larcker criterion, the square root of AVE for each latent variable was greater than its correlations with other variables. All HTMT ratios were below 0.85, with the maximum value being 0.580, indicating satisfactory discriminant validity. Overall, the measurement model showed acceptable reliability and validity for subsequent structural model analysis. Nevertheless, because several constructs are conceptually related and all indicators are self-reported, conceptual overlap should still be interpreted cautiously.

**Table 2:** Measurement model evaluation.

Latent variable	Indicator	Loading	VIF	CA	CR	AVE
ECL	ECL1	0.911	3.419	0.931	0.951	0.828
ECL	ECL2	0.911	3.416			
ECL	ECL3	0.907	3.362			
ECL	ECL4	0.911	3.41			
ICL	ICL1	0.903	3.085	0.914	0.939	0.795
ICL	ICL2	0.905	3.153			
ICL	ICL3	0.881	2.671			
ICL	ICL4	0.876	2.548			
IQ	IQ1	0.879	2.504	0.894	0.926	0.759
IQ	IQ2	0.867	2.392			
IQ	IQ3	0.859	2.259			
IQ	IQ4	0.879	2.59			
PCK	PCK1	0.873	2.605	0.899	0.93	0.767
PCK	PCK2	0.86	2.421			
PCK	PCK3	0.888	2.61			
PCK	PCK4	0.883	2.536			
PEOU	PEOU1	0.843	2.144	0.889	0.923	0.75
PEOU	PEOU2	0.87	2.388			
PEOU	PEOU3	0.88	2.63			
PEOU	PEOU4	0.869	2.28			
PINT	PINT1	0.845	2.216	0.882	0.919	0.738
PINT	PINT2	0.847	2.053			
PINT	PINT3	0.858	2.296			
PINT	PINT4	0.887	2.549			
SLI	SLI1	0.869	2.385	0.895	0.927	0.759
SLI	SLI2	0.868	2.452			
SLI	SLI3	0.867	2.39			
SLI	SLI4	0.882	2.47			

CA, Cronbach's alpha; CR, composite reliability; AVE, average variance extracted; VIF, variance inflation factor. All constructs were specified as reflective. VIF values were examined as an additional collinearity diagnostic.

**Table 3a:** Fornell–Larcker criterion.

Variable	ECL	ICL	IQ	PCK	PEOU	PINT	SLI
ECL	0.910						
ICL	0.309	0.892					
IQ	−0.340	−0.468	0.871				
PCK	−0.176	−0.527	0.367	0.876			
PEOU	−0.346	−0.484	0.393	0.427	0.866		
PINT	0.421	0.381	−0.406	−0.313	−0.365	0.859	
SLI	−0.262	−0.476	0.409	0.390	0.401	−0.349	0.871

Diagonal values represent the square roots of AVE.

**Table 3b:** HTMT ratios.

Variable	ECL	ICL	IQ	PCK	PEOU	PINT
ICL	0.334					
IQ	0.372	0.518				
PCK	0.191	0.580	0.409			
PEOU	0.379	0.535	0.439	0.474		
PINT	0.462	0.422	0.454	0.347	0.409	
SLI	0.286	0.525	0.456	0.431	0.446	0.388

All HTMT values are below 0.85.

#### 4.1.3 Structural model evaluation

**Figure 3:**
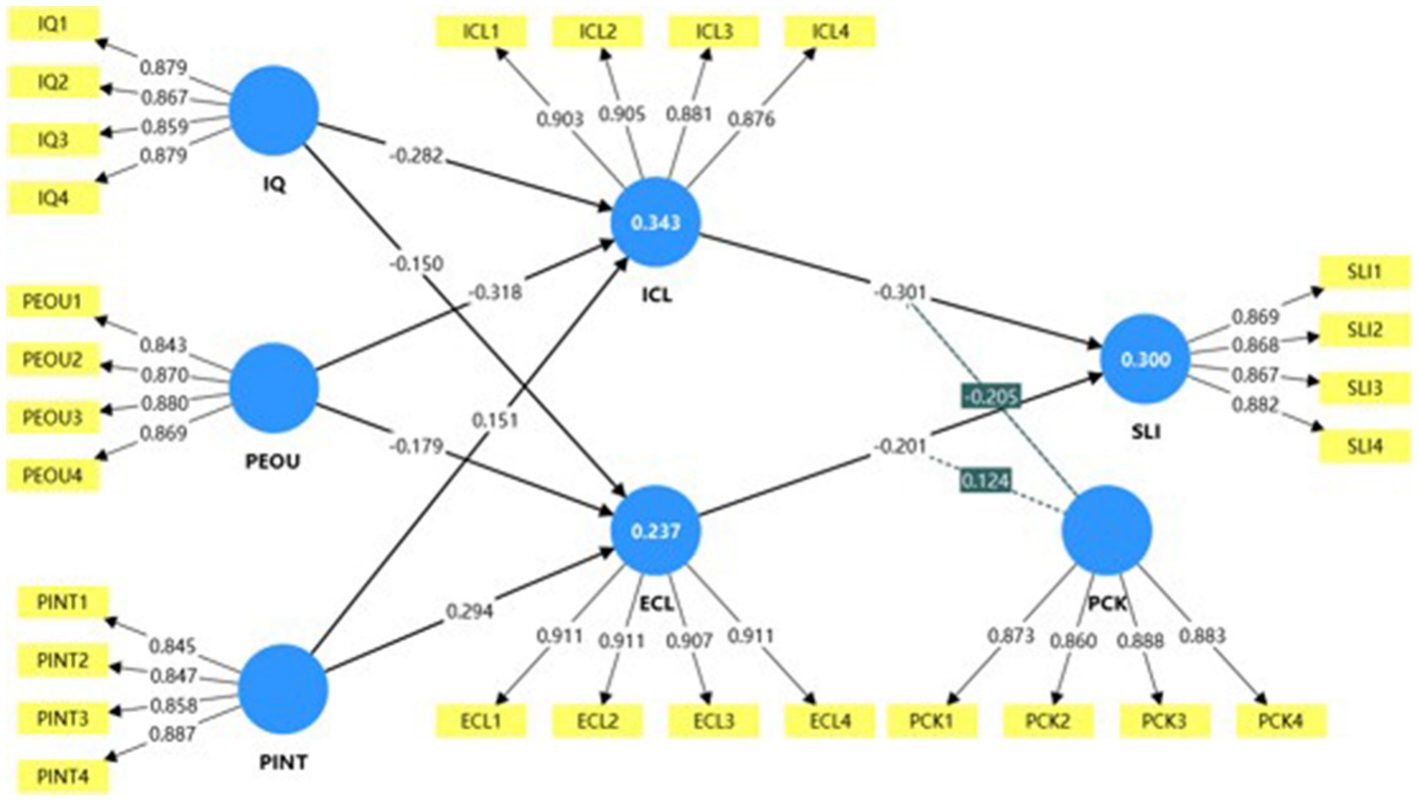
Structural model.

After the measurement model was validated, SmartPLS 4.1 was used to estimate the structural model, and 5,000 bootstrap resamples were used to test the significance of path coefficients. As shown in Figure 3, the overall path relationships are clear. As shown in Table 4, the *R*^2^ values for intrinsic cognitive load, extraneous cognitive load, and sustained learning intention were 0.343, 0.237, and 0.300, respectively, and the corresponding Q^2^ values were all greater than 0. These results indicate acceptable explanatory relevance, with moderate explanatory power for sustained learning intention. In terms of global fit, SRMR was 0.062 and NFI was 0.886, which are acceptable for reporting in PLS-SEM but should be interpreted cautiously rather than as evidence of strong model fit.

**Table 4:** Explanatory power and model fit indicators.

Indicator	Variable/Path	ECL	ICL	SLI	Overall
*R* ^2^	—	0.237	0.343	0.300	—
*Q* ^2^	—	0.225	0.333	0.251	—
*f* ^2^	ECL	—	—	0.044	—
*f* ^2^	ICL	—	—	0.083	—
*f* ^2^	IQ	0.023	0.093	—	—
*f* ^2^	PCK	—	—	0.060	—
*f* ^2^	PEOU	0.033	0.122	—	—
*f* ^2^	PINT	0.089	0.027	—	—
SRMR	—	—	—	—	0.062
NFI	—	—	—	—	0.886

As shown in Table 5, information quality was significantly negatively associated with intrinsic cognitive load (β = −0.282, *p* < 0.001) and extraneous cognitive load (β = −0.150, *p* = 0.001). Perceived ease of use was also significantly negatively associated with intrinsic cognitive load (β = −0.318, *p* < 0.001) and extraneous cognitive load (β = −0.179, *p* < 0.001), indicating that high-quality information and higher ease of use were linked to lower perceived cognitive burden in digital cultural learning. Contrary to the hypotheses, perceived interactivity showed significant positive associations with both intrinsic cognitive load (β = 0.151, *p* = 0.001) and extraneous cognitive load (β = 0.294, *p* < 0.001), suggesting that stronger perceived interactivity was linked to greater processing pressure in this context. Intrinsic cognitive load (β = −0.301, *p* < 0.001) and extraneous cognitive load (β = −0.201, *p* < 0.001) were both significantly negatively associated with sustained learning intention. Overall, information quality and perceived ease of use were associated with sustained learning intention mainly through lower cognitive load, whereas perceived interactivity showed an association pattern opposite to the original hypotheses.

**Table 5:** Direct-effect results.

Hypothesis	Path	β	STDEV	*T*	*P*	2.5%	97.5%	Result
H1a	IQ → ICL	−0.282	0.046	6.181	< 0.001	−0.372	−0.194	Supported
H1b	IQ → ECL	−0.150	0.044	3.397	0.001	−0.239	−0.066	Supported
H2a	PEOU → ICL	−0.318	0.044	7.240	< 0.001	−0.406	−0.234	Supported
H2b	PEOU → ECL	−0.179	0.044	4.057	< 0.001	−0.267	−0.095	Supported
H3a	PINT → ICL	0.151	0.046	3.293	0.001	0.064	0.245	Opposite to hypothesis
H3b	PINT → ECL	0.294	0.048	6.185	< 0.001	0.200	0.387	Opposite to hypothesis
H4	ICL → SLI	−0.301	0.054	5.580	< 0.001	−0.404	−0.193	Supported
H5	ECL → SLI	−0.201	0.044	4.549	< 0.001	−0.285	−0.114	Supported

#### 4.1.4 Indirect-effect test

Based on the direct-path results, the indirect associations of cognitive load between external support cues and sustained learning intention were further examined. As shown in Table 6, information quality and perceived ease of use showed significant positive indirect associations with sustained learning intention through lower intrinsic and extraneous cognitive load. In contrast, perceived interactivity showed significant negative indirect associations with sustained learning intention through higher levels of both types of cognitive load. The indirect associations through intrinsic cognitive load were generally stronger, indicating that intrinsic cognitive load may play a more prominent statistical transmission role in the association between external support cues and sustained learning intention.

**Table 6:** Statistical indirect association results.

Path	β	STDEV	*T*	*P*	2.5%	97.5%
IQ → ICL → SLI	0.085	0.021	3.979	< 0.001	0.050	0.136
IQ → ECL → SLI	0.030	0.012	2.602	0.009	0.012	0.057
PEOU → ICL → SLI	0.096	0.022	4.299	< 0.001	0.057	0.145
PEOU → ECL → SLI	0.036	0.013	2.873	0.004	0.016	0.066
PINT → ICL → SLI	−0.045	0.016	2.773	0.006	−0.085	−0.019
PINT → ECL → SLI	−0.059	0.017	3.579	< 0.001	−0.096	−0.031

#### 4.1.5 Moderation test

The moderating role of prior cultural knowledge in the associations between cognitive load and sustained learning intention was then examined. As shown in Table 7, the interaction term PCK × ICL → SLI was significantly negative (β = −0.205, *p* < 0.001), indicating that higher prior cultural knowledge was associated with a stronger negative relationship between intrinsic cognitive load and sustained learning intention; thus, H6a was not supported. By contrast, the interaction term PCK × ECL → SLI was significantly positive (β = 0.124, *p* = 0.013), indicating that higher prior cultural knowledge was associated with a weaker negative relationship between extraneous cognitive load and sustained learning intention; thus, H6b was supported. This pattern suggests that prior cultural knowledge is more strongly associated with buffering avoidable burden caused by information organization and interaction design than with reducing the necessary processing associated with content complexity itself.

**Table 7:** Moderation-effect results.

Hypothesis	Path	β	STDEV	*T*	*P*	2.5%	97.5%	Result
H6a	PCK × ICL → SLI	−0.205	0.053	3.879	< 0.001	−0.305	−0.094	Not supported
H6b	PCK × ECL → SLI	0.124	0.050	2.493	0.013	0.017	0.212	Supported

Results are based on bootstrap confidence intervals.

**Figure 4:**
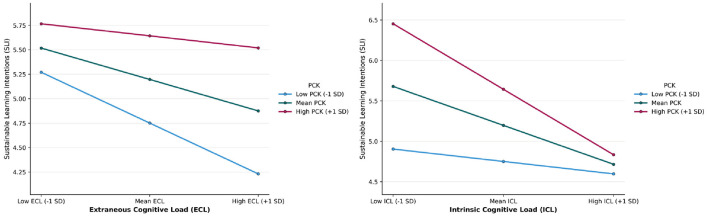
Simple-slope plots for the moderating effects of prior cultural knowledge on the relationships between cognitive load and sustained learning intention.

To facilitate interpretation, the two moderation effects are further illustrated using simple-slope plots at low, medium, and high levels of prior cultural knowledge (Figure 4). Low, medium, and high prior cultural knowledge correspond to −1 SD, the mean, and +1 SD, respectively.

### 4.2 fsQCA Results

#### 4.2.1 Variable calibration

In this study, the outcome and antecedent conditions were treated as sets, and the data for each case were transformed into fuzzy-set membership scores through direct calibration. Following prior fsQCA research and the distributional characteristics of seven-point Likert-scale data, the 75th, 50th, and 25th percentiles of the sample distribution were used as the anchors for full membership, crossover point, and full non-membership, respectively. A value calibrated exactly at 0.500 was adjusted to 0.501 to avoid threshold ambiguity in fuzzy-set analysis. Percentile-based calibration was adopted because the present study focuses on relative set membership in perception-based learning data rather than on externally fixed substantive cutoffs. The detailed calibration anchors are reported in Appendix B.

#### 4.2.2 Necessary-condition analysis

After calibration, each antecedent condition was examined to determine whether it constituted a necessary condition for the outcome. As shown in Table 8, the consistency of each single antecedent condition for high or low sustained learning intention did not reach the strict 0.90 threshold. Therefore, no single condition independently constituted a necessary condition for high SLI or low SLI. This indicates that sustained learning intention is not determined by any single factor but is more likely produced by the joint effects of multiple conditions.

**Table 8:** Necessary-condition analysis.

Condition	High SLI consistency	High SLI coverage	Low SLI consistency	Low SLI coverage
IQ	0.601	0.607	0.531	0.494
~IQ	0.499	0.535	0.578	0.572
PEOU	0.572	0.594	0.558	0.535
~PEOU	0.552	0.575	0.577	0.554
PINT	0.564	0.572	0.578	0.541
~PINT	0.548	0.585	0.543	0.535
ICL	0.446	0.458	0.712	0.675
~ICL	0.683	0.720	0.428	0.416
ECL	0.438	0.449	0.681	0.643
~ECL	0.652	0.689	0.417	0.407
PCK	0.722	0.689	0.469	0.412
~PCK	0.384	0.439	0.647	0.682

#### 4.2.3 Configurational path analysis

As shown in Table 9, five configurations leading to high sustained learning intention were identified. Their consistency ranged from 0.870 to 0.912, with an overall solution consistency of 0.868 and an overall solution coverage of 0.408, indicating that these pathways explain approximately 40.8% of high-SLI cases. Overall, information quality and prior cultural knowledge more frequently appeared as core or key conditions, while intrinsic and extraneous cognitive load were often absent or compensated for by other positive conditions in specific contexts. This indicates that high sustained learning intention is not driven by a single factor but depends more on combinations of high-quality information support, lower cognitive burden, and sufficient knowledge foundations. Specifically, H1 represents a high-information-quality and low-extraneous-load pathway; H2 can be described as an information-interaction-knowledge synergy pathway; H3 is a high-ease-of-use, high-interactivity, and low-load pathway; H4 is a high-interactivity and knowledge-compensation pathway, showing that even when perceived ease of use is low, strong interaction and high prior cultural knowledge may still produce high SLI; and H5 is an information-ease-of-use-knowledge compensation pathway, showing that when high information quality, high perceived ease of use, and high prior cultural knowledge coexist, the adverse effect of extraneous cognitive load may be partially offset. Three configurations leading to low sustained learning intention were also identified, with an overall solution consistency of 0.866 and an overall coverage of 0.352. Intrinsic and extraneous cognitive load more frequently appeared as core or key conditions, whereas information quality, perceived ease of use, and prior cultural knowledge were more often absent or insufficient. This suggests that learners are more likely to show low subsequent engagement when positive external support is insufficient and cognitive burden is high. L1 can be summarized as a high-load and low-knowledge pathway; L2 is a high-load and low-ease-of-use pathway, showing that even when information quality is high, low perceived ease of use and high cognitive load may suppress SLI; and L3 is a low-information and high-load pathway, emphasizing that insufficient information support, weak knowledge foundations, and high cognitive burden jointly lead to low SLI. These configurational pathways should be interpreted as context-specific patterns rather than deterministic practical recipes, especially because overall solution coverage is moderate and several pathways show relatively low unique coverage.

**Table 9:** Configurational pathways for sustained learning intention.

Condition	H1	H2	H3	H4	H5	L1	L2	L3
IQ	●	•			●		●	⊗
PEOU	⊗		●	⊗	•		⊗	•
PINT		•	●	•		●		
ICL		⊘	⊗	●	⊗	•	•	•
ECL	⊗		⊗	⊗	●	●	●	●
PCK	•	•	●	●	•	⊗	⊗	⊗
Consistency	0.912	0.870	0.881	0.876	0.882	0.873	0.845	0.894
Raw coverage	0.181	0.215	0.170	0.105	0.138	0.278	0.127	0.119
Unique coverage	0.055	0.024	0.023	0.037	0.049	0.157	0.030	0.028
Overall solution consistency	0.868					0.866		
Overall solution coverage	0.408					0.352		

●indicates the presence of a core condition; ⊗ indicates the absence of a core condition; • indicates the presence of a peripheral condition; ⊘ indicates the absence of a peripheral condition. Blank cells indicate that the condition may be either present or absent.

#### 4.2.4 Robustness test

To examine the robustness of the fsQCA results, the raw consistency threshold was increased from 0.80 to 0.85, and the case-frequency threshold was increased from 5 to 6. Under these stricter settings, the high-SLI configurations remained substantively consistent with the original solution. For low SLI, the stricter solution converged from three configurations to two configurations that corresponded substantively to subsets of the original solution. No contradictory substantive conclusion emerged. These results indicate that the configurational findings were not substantially affected by reasonable changes in the consistency and case-frequency thresholds and therefore showed acceptable robustness. The detailed robustness-check solution is reported in Appendix H.

## 5 Discussion

### 5.1 Cognitive manageability as the core educational psychology mechanism of sustained learning

The core finding of this study is that, within a heterogeneous generative-AI-supported digital cultural learning context, learners' self-reported sustained learning intention was associated not simply with whether more support was perceived to be available, but with whether such support was perceived as cognitively manageable. To examine this issue, this study analyzed an explanatory framework linking perceived external support cues, perceived cognitive load, and self-reported sustained learning intention. The overall results suggest that sustained learning intention should not be interpreted as a direct educational effect of generative AI itself, but as an association pattern among learners' perceptions of AI support, cognitive burden, and post-experience learning willingness. In other words, in the present digital cultural learning context, the key issue is not whether AI support exists in general, but whether learners perceive that support as cognitively manageable, maintainable, and continuously absorbable (Pescapè, 2024; Granda et al., 2024). These findings should therefore be interpreted as perception-based associations observed in one heterogeneous generative-AI-supported digital cultural learning context rather than as general causal effects of AI-supported learning.

### 5.2 How high-quality, low-friction support is transformed into sustained learning intention

In relation to RQ1 and RQ2, the results first show that information quality and perceived ease of use were significantly negatively associated with both intrinsic and extraneous cognitive load, while both types of cognitive load were significantly negatively associated with sustained learning intention. In digital cultural learning contexts characterized by high information density, long interpretive chains, and complex conceptual layers, the educational value of generative AI lies not mainly in whether it is more intelligent or advanced, but in whether it supports understanding in a clear, low-effort, and low-interference manner. Information quality may help reduce perceived ambiguity, redundancy, and irrelevant processing, thereby supporting more stable content judgments. Perceived ease of use may lower the perceived costs of interface understanding, path search, and operational control, allowing learners to allocate more cognitive resources to cultural content than to the tool itself (Calisir and Calisir, 2004; Cho et al., 2009). Thus, cognitively manageable support from generative AI appears to be associated with lower cognitive friction rather than being directly produced by functional supply. Theoretically, this result supports the hierarchical logic of S-O-R in this study and demonstrates that cognitive load is the key mechanism linking external support and subsequent learning engagement.

This finding has implications for understanding perceived AI support in the present digital cultural learning context. High-quality, low-friction support appeared to be associated with stronger sustained learning intention because learners reported a greater willingness to continue learning when information was clear and interaction paths were cognitively manageable (Bozkurt et al., 2023). Therefore, the value of generative AI in this study should be understood within this specific context: it does not demonstrate the general educational effect of generative AI, but shows that learners' perceived information quality and perceived ease of use were linked to sustained learning intention mainly through lower perceived cognitive burden. This result is broadly consistent with prior findings on information quality, perceived ease of use, and sustained learning outcomes, while further indicating that, in this dataset, these variables were associated with sustained learning intention mainly through the cognitive-load mechanism rather than through a direct pathway (Zheng et al., 2023; Guo et al., 2025).

### 5.3 When perceived interactivity becomes cognitive processing demand

The most noteworthy result is that perceived interactivity did not reduce cognitive load as hypothesized, but significantly increased both intrinsic and extraneous cognitive load. This should not be treated as an anomalous result; rather, it represents the most important theoretical increment of this study. It shows that stronger human-AI interaction does not automatically imply higher-quality learning support in generative AI-supported digital cultural learning. For traditional cultural content such as Mawangdui, which is information-intensive and semantically dependent, interaction means not only more timely responses but also multi-round questioning, extended interpretive chains, increased information switching, and frequent feedback reorganization. The stronger the interaction, the more learners may need to compare, select, and integrate among different explanatory paths, thereby assuming new processing costs while receiving support (Rosé et al., 2019). Interaction is therefore not an unconditionally positive resource but a threshold-like double-edged condition. When controlled within learners' manageable range, it can support understanding; when it exceeds cognitive tolerance, it may shift from support to burden.

This finding offers a context-bound supplement to the assumption that stronger interaction necessarily supports better learning outcomes (Arnold et al., 2024). In the present digital cultural learning context, the issue is not stronger human-AI interaction in general, but whether interaction is perceived as cognitively manageable by learners. Interaction frequency and feedback intensity may be useful only when they are organized with an appropriate explanatory rhythm, clear information hierarchy, and manageable cognitive demands. Therefore, the reverse results for H3a and H3b should be interpreted as a perception-based finding within a high-complexity cultural learning context rather than as a general conclusion about AI interactivity. The present context is not general information search or a low-complexity task, but digital cultural learning characterized by high information density, strong semantic dependence, and long interpretive chains. In this setting, increased perceived interactivity may accompany more follow-up questions, more frequent information switching, and more complex explanatory branches. Its association with cognitive load therefore appears conditional on learners' perceived cognitive capacity. This offers a context-bound supplement to prior findings that interactivity is associated with engagement and perceived cultural value (Meng and Dolah, 2025; Wu et al., 2024; Ren et al., 2025). At the same time, alternative explanations such as novelty effects, self-selection into AI use, response style, prior AI experience, and residual common method variance cannot be fully ruled out in the present cross-sectional design.

### 5.4 Prior knowledge as a differential cognitive boundary condition

In relation to RQ2, this study further finds that the moderating effects of prior cultural knowledge differ across the two types of cognitive load. Higher prior cultural knowledge was associated with a weaker negative relationship between extraneous cognitive load and sustained learning intention, but it did not show the same buffering pattern for intrinsic cognitive load. Instead, higher prior cultural knowledge was associated with a stronger negative relationship between perceived intrinsic cognitive burden and sustained learning intention. This indicates that prior cultural knowledge is not a universal load reducer but a boundary condition that operates differently depending on load source. For extraneous cognitive load caused by interface organization, redundant feedback, and complex interaction paths, higher prior cultural knowledge helps learners identify key content more quickly, filter distracting information, and maintain understanding fluency through existing schemas. It therefore has a clear buffering effect. By contrast, for intrinsic cognitive load caused by content complexity, prior cultural knowledge does not necessarily make the task easier. It may activate more existing schemas, comparative processing, and semantic discrimination, making learners more sensitive to historical relations, conceptual layers, and interpretive details. As a result, it may deepen necessary processing rather than reduce its intensity. In other words, higher prior cultural knowledge mainly buffers avoidable extra burden, not the unavoidable complexity inherent in the content itself (Jegede and Aikenhead, 1999).

This finding should also be interpreted in light of the conceptual status of the ICL construct in this study. Although the construct was grounded in cognitive load theory, it was measured as learners' perceived intrinsic cognitive burden in a semantically dense cultural learning context. Therefore, it may partly capture learners' perceived difficulty, cognitive effort, and depth of semantic processing, rather than intrinsic load in a strictly objective task-inherent sense. This distinction is particularly relevant for interpreting the moderation result. Learners with higher prior cultural knowledge may notice more historical relations, symbolic meanings, and interpretive details, which may make the cultural content feel more cognitively demanding even though their schemas help them process avoidable external burden. Thus, the negative moderation pattern for PCK × ICL should be interpreted cautiously as a finding about perceived intrinsic cognitive burden, not as evidence that prior knowledge necessarily increases objective intrinsic load.

The importance of this finding lies in redefining knowledge foundation from a generally positive resource to a cognitive boundary condition with differentiated effects on different load types. Generative AI-assisted learning design should therefore not simply classify learners as having more or less knowledge, but should distinguish whether they face avoidable extra burden or unavoidable content complexity. The former is better addressed through layered explanations, interface optimization, and concise feedback, whereas the latter requires conceptual progression, background links, and challenge management. Personalized support for different learners should not remain at the level of individual preference; it should be implemented as differentiated responses to different sources of cognitive load. This finding also suggests that prior knowledge does not universally reduce load but instead acts as a cognitive boundary condition that regulates different load sources in different ways (Zheng et al., 2023; Huangfu et al., 2026).

### 5.5 From net effects to configurational pathways: heterogeneous formation of high and low sustained learning intention

In relation to RQ3, the fsQCA results further show that sustained learning intention is not determined by a single factor, but is a configurational outcome jointly formed by external support cues, cognitive load states, and knowledge foundation. The necessary-condition analysis shows that no single condition is sufficient to constitute high or low SLI, while the configurational analysis identifies five high-SLI pathways and three low-SLI pathways. Overall, high SLI more often co-occurs with positive conditions such as information quality, perceived ease of use, and prior cultural knowledge, whereas low SLI is more often accompanied by the presence of intrinsic and extraneous cognitive load and the absence of prior cultural knowledge. This means that sustained learning intention is not the result of the linear increase or decrease of one core variable, but of synergy, compensation, and substitution among multiple conditions. Some high-SLI pathways further show that even when extraneous cognitive load remains present, its adverse effect may be partially offset if high information quality, high perceived ease of use, and high prior cultural knowledge coexist. This again indicates that high sustained learning intention is closer to condition balance than to single-cause reinforcement.

**Figure 5:**
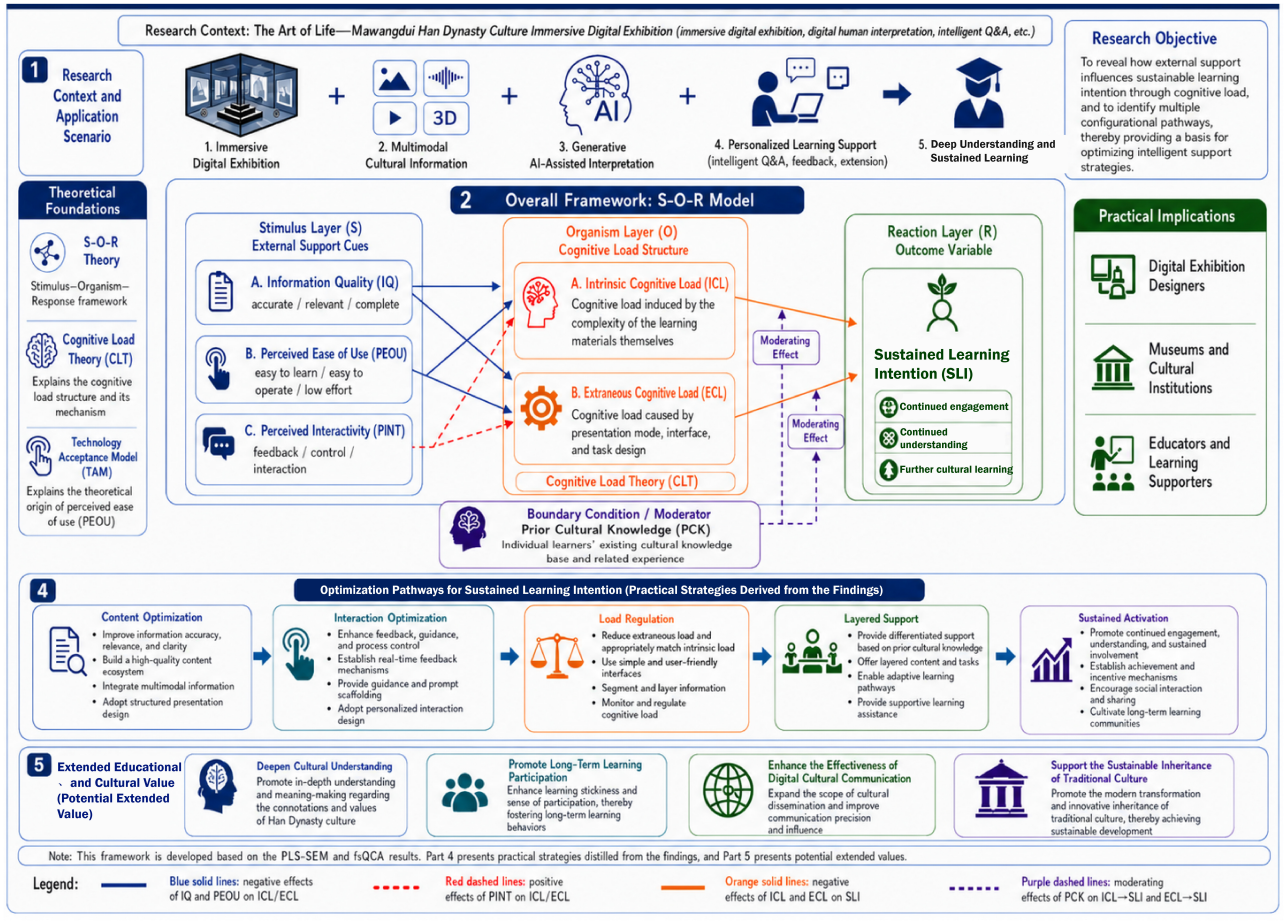
Conceptual synthesis framework of sustained learning intention in generative AI-supported digital cultural learning. This framework is a conceptual synthesis derived from the PLS-SEM and fsQCA findings. It is intended to organize the empirical results and practical implications, rather than to present an additional empirically tested or validated causal model.

The relationship between PLS-SEM and fsQCA is complementary rather than mechanical. PLS-SEM answers which mechanisms operate most stably on average and thus reveals dominant paths among information quality, perceived ease of use, perceived interactivity, and cognitive load. fsQCA answers how these mechanisms occur not through a single path but through different condition combinations that produce similar or opposite outcomes. It therefore explains the heterogeneity and configurational asymmetry in sustained learning intention formation. Only by combining the two methods can this study explain both why sustained learning intention emerges and through which different pathways it may be formed. This constitutes the most substantive methodological contribution of the study. The findings also show that sustained learning outcomes in generative AI-assisted contexts are mechanistically heterogeneous and are consistent with prior research on configurational characteristics and configurational asymmetry in complex learning outcomes (Zheng et al., 2023). To further synthesize the PLS-SEM and fsQCA findings, this study presents Figure 5 as a conceptual synthesis framework for interpreting sustained learning intention in generative AI-supported digital cultural learning. This framework is not proposed as an additional statistically tested model. To avoid overinterpretation, Figure 5 should be read as a conceptual synthesis and practical interpretation of the empirical findings, not as a validated causal structure. The arrows and pathways in the framework summarize the theoretical interpretation of the PLS-SEM and fsQCA results and should not be understood as additional hypotheses tested beyond the reported statistical analyses. Rather, the framework organizes the main empirical patterns and practical implications, including content optimization, interaction optimization, load regulation, layered support, and sustained activation.

Within this conceptual synthesis, sustained learning intention is interpreted as an outcome associated with cognitively mediated support rather than as a direct consequence of stronger technological stimulation. Information quality and perceived ease of use are positioned as low-friction support cues that are negatively associated with cognitive load, whereas perceived interactivity is presented as a double-edged condition that may increase processing demands when interaction becomes excessive, fragmented, or poorly structured. Prior cultural knowledge is further treated as a boundary condition that differentially moderates the relationships between cognitive load and sustained learning intention. Accordingly, the practical implications of the model should not be understood as a call for more interaction or more functions, but as a call for cognitively manageable AI-supported learning design.

### 5.6 Theoretical contributions and practical implications

The main theoretical contribution of this study is to provide a perception-based account of how learners' reported AI support experiences are associated with sustained learning intention in a heterogeneous digital cultural learning context. Rather than demonstrating the educational effects of generative AI itself, the study shows that learners' perceived external support cues were linked to self-reported post-learning engagement through perceived cognitive burden. Information quality and perceived ease of use were associated with lower cognitive friction, whereas perceived interactivity was associated with higher processing demand in this particular context. Prior cultural knowledge further operated as a differential boundary condition rather than as a universal load reducer. Taken together, these findings frame sustained learning intention as a psychologically mediated perception-based outcome within naturally occurring AI-supported cultural learning experiences, rather than as a direct extension of technology use or as evidence of general AI learning effects.

### 5.7 Limitations and future research

This study has several limitations. First, it is based on cross-sectional questionnaire data and is therefore more suitable for explaining structural associations among variables than for making strong causal claims. Second, all main constructs, including cognitive load, prior cultural knowledge, and sustained learning intention, were measured through self-report, which means that residual common method variance and response-style effects cannot be fully ruled out. Third, sustained learning intention was measured as a self-reported intention rather than as observed long-term learning behavior or objective learning outcomes. Fourth, the actual generative AI exposure was not fully standardized across participants, because respondents reported their most recent and most complete AI-supported cultural learning experience rather than participating in one experimentally controlled AI intervention. Another limitation is that detailed prior AI experience was not retained as an independent background variable in the final analytical dataset. Although all valid respondents reported relevant generative-AI-supported cultural learning experience, learners' broader familiarity with generative AI may still have influenced their perceived ease of use, perceived interactivity, cognitive load, and sustained learning intention simultaneously. Therefore, the observed associations may partly reflect differences in prior AI experience. Future studies should measure prior AI familiarity, frequency of AI use, AI literacy, and domain-specific AI experience more directly and examine their potential confounding or moderating roles.

Fifth, the study did not include objective cognitive load indicators, process data, or a control group. Sixth, the ICL construct should be interpreted as perceived intrinsic cognitive burden in a semantically dense cultural learning context rather than as a purely objective task-inherent load indicator. Seventh, although procedural and statistical remedies were applied, potential common method bias cannot be completely excluded. Finally, because the study focused on one Chinese digital cultural learning context, namely the Mawangdui immersive digital exhibition, the generalizability of the findings to other cultural topics, AI tools, and learner populations remains limited.

In addition, the findings should not be directly generalized to highly technical or accuracy-sensitive learning domains such as STEM education. The present study was conducted in a digital cultural learning context, where generative AI mainly supported interpretation, explanation, cultural association, and meaning construction. In contrast, STEM learning often involves stricter requirements for procedural accuracy, symbolic reasoning, calculation, and problem-solving precision. In such contexts, AI-generated errors or hallucinations may not only increase extraneous cognitive load but also lead to conceptual confusion, frustration, or incorrect problem-solving strategies. Therefore, the cognitive-load mechanism identified in this study may operate differently in technical subjects such as mathematics, physics, engineering, or computer science. Future research should examine whether the relationships among generative AI support, cognitive load, prior knowledge, and sustained learning intention differ across cultural, interpretive, and technical learning domains. Future research may combine longitudinal, experimental, process-based, and multi-context designs to test these mechanisms more rigorously, especially by comparing cultural, interpretive, and technical learning domains.

## 6 Conclusion

This study examined how learners' perceived generative AI support was associated with self-reported sustained learning intention through perceived cognitive load mechanisms in a heterogeneous digital cultural learning context. Using the Mawangdui Han Culture Immersive Digital Exhibition as the focal context, the study analyzed an explanatory framework linking perceived AI support cues, perceived cognitive load, prior cultural knowledge, and sustained learning intention.

The findings provide three main conclusions within the boundary of this cross-sectional, perception-based survey. First, information quality and perceived ease of use were negatively associated with intrinsic and extraneous cognitive load, whereas perceived interactivity was positively associated with both forms of load. This suggests that, in this dataset, the perceived value of AI support was related less to stronger stimulation or more interaction and more to whether learners experienced the learning process as clear, low-effort, and low-interference. Second, intrinsic and extraneous cognitive load were both negatively associated with sustained learning intention. Prior cultural knowledge weakened only the negative association between extraneous cognitive load and sustained learning intention, but strengthened the negative association between perceived intrinsic cognitive burden and sustained learning intention. This result should be interpreted cautiously because ICL was measured as perceived intrinsic cognitive burden rather than as objective task-inherent load. Third, the fsQCA results indicate that high and low sustained learning intention were associated with multiple configurations of perceived AI support features, perceived cognitive load states, and prior knowledge.

Overall, the study suggests that, in naturally occurring and heterogeneous generative-AI-supported digital cultural learning experiences, AI support should be evaluated in terms of whether it is perceived as cognitively manageable by learners. The findings should not be interpreted as evidence of the general educational effects of generative AI itself, but as evidence of associations among learner perceptions within a specific digital cultural learning context. For learning design in similar contexts, AI-supported systems may be more helpful when they function as low-friction, low-redundancy, and adaptive instructional support rather than as tools that simply increase interaction intensity or information supply.

## Data Availability

The raw data supporting the conclusions of this article will be made available by the authors, without undue reservation.

## References

[B1] Almahamid S. Mcadams A. C. Taher A. K. Mo'taz A.-S. E. (2010). The relationship between perceived usefulness, perceived ease of use, perceived information quality, and intention to use e-government. J. Theor. Appl. Inf. Technol. 11, 30–44.

[B2] Al-Mamary Y. H. Shamsuddin A. Aziati N. (2014). The relationship between system quality, information quality, and organizational performance. Int. J. Knowl. Res. Manag. E-Commerce 4, 7–10.

[B3] Alwakid W. N. Dahri N. A. Humayun M. Alwakid G. N. Integrating A. I. (2025). chatbots for enhancing academic support in business education: a SEM-based study toward sustainable learning. Int. J. Manag. Educ. 23:101252. doi: 10.1016/j.ijme.2025.101252

[B4] Arnold O. Hölzer W. Jantke K. (2024). Generative, AI for education, research and discovery: issues of conjectures and refutations. J. Contemp. Educ. Theory Artif. Intell. 1: JCETAI-109. doi: 10.71010/jcetai-2024-e109

[B5] Bakhnoo M. Rostamzadeh R. Babazadeh Sangar A. Sarhangi K. (2026). Balancing human interaction and system digitization in the phygital ecosystem: a metasynthesis study. Hum. Behav. Emerg. Technol. 2026:6675139. doi: 10.1155/hbe2/6675139

[B6] Ben-Eliyahu A. (2021). Sustainable learning in education. Sustainability 13:4250. doi: 10.3390/su13084250

[B7] Bozkurt A. Xiao J. Lambert S. Pazurek A. Crompton H. Koseoglu S. . (2023). Speculative futures on ChatGPT and generative artificial intelligence (AI): a collective reflection from the educational landscape. Asian J. Distance Educ. 18, 53–130. doi: 10.5281/zenodo.7636568

[B8] Cai Z. Liu C. Yang Y. Li B. (2025). The impact of learning supports in digital game-based learning on learners with different levels of prior knowledge. Internet Higher Educ. 68:101044. doi: 10.1016/j.iheduc.2025.101044

[B9] Calisir F. Calisir F. (2004). The relation of interface usability characteristics, perceived usefulness, and perceived ease of use to end-user satisfaction with enterprise resource planning (ERP) systems. Comput. Hum. Behav. 20, 505–515. doi: 10.1016/j.chb.2003.10.004

[B10] Cheng Y-. M. (2014). Roles of interactivity and usage experience in e-learning acceptance: a longitudinal study. Int. J. Web Inf. Syst. 10, 2–23. doi: 10.1108/IJWIS-05-2013-0015

[B11] Cho V. Cheng T. E. Lai W. J. (2009). The role of perceived user-interface design in continued usage intention of self-paced e-learning tools. Comput. Educ. 53, 216–227. doi: 10.1016/j.compedu.2009.01.014

[B12] Davis F. D. (1989). Perceived usefulness, perceived ease of use, and user acceptance of information technology. MIS Q. 13, 319–340. doi: 10.2307/249008

[B13] Dong Q. He J. Li N. Wang B. Lu H. Yang Y. . (2025). Exploring the cognitive reconstruction mechanism of generative ai in outcome-based design education: a study on load optimization and performance impact based on dual-path teaching. Buildings 15:2864. doi: 10.3390/buildings15162864

[B14] Fassott G. Henseler J. Coelho P. S. (2016). Testing moderating effects in PLS path models with composite variables. Ind. Manag. Data Syst. 116, 1887–1900. doi: 10.1108/IMDS-06-2016-0248

[B15] Fu S. Gu H. Yang B. (2020). The affordances of AI-enabled automatic scoring applications on learners' continuous learning intention: an empirical study in China. Br. J. Educ. Technol. 51, 1674–1692. doi: 10.1111/bjet.12995

[B16] GorbunovaA. KapuzaA. ChenO. CostleyJ. (2025). Rethinking pre-training: cognitive load implications for learners with varying prior knowledge. Front. Psychol. 16:1628047. doi: 10.3389/fpsyg.2025.162804740851629 PMC12367772

[B17] Gorbunova A. Lange C. Savelyev A. Adamovich K. Costley J. (2024). The interplay of self-regulated learning, cognitive load, and performance in learner-controlled environments. Educ. Sci. 14:860. doi: 10.3390/educsci14080860

[B18] Granda B. S. Inzhivotkina Y. Apolo M. F. I. Fajardo J. G. U. (2024). Educational innovation: exploring the potential of generative artificial intelligence in cognitive schema building. Edutec, Revista Electrónica de Tecnología Educativa 89, 44–63. doi: 10.21556/edutec.2024.89.3251

[B19] Grunwald G. Kara A. Spillan J. E. (2025). Fuzzy set qualitative comparative analysis (fsQCA) to examine causal complexity in business students' sustainability behavioural intentions. Higher Educ. Q. 79:e70076. doi: 10.1111/hequ.70076

[B20] Guo J. Ren H. Qi Y. (2025). Exploring factors influencing students' sustainable learning intention in the application of augmented reality in education: a case study in radio and television scripting and directing. Interact. Learn. Environ. 33, 5726–5746. doi: 10.1080/10494820.2025.2487515

[B21] Gupta P. Prashar S. Vijay T. S. Parsad C. (2021). Examining the influence of antecedents of continuous intention to use an informational app: the role of perceived usefulness and perceived ease of use. Int. J. Bus. Inf. Syst. 36, 270–287. doi: 10.1504/IJBIS.2021.112829

[B22] Hao X. Xu J. Wang Y. (2025). How generative AI shapes user perceived value and adoption intention in digital museum experiences. npj Heritage Sci. 13:608. doi: 10.1038/s40494-025-02194-9

[B23] He X. Wu D. (2023). Does additional audio really work? A study on users' cognitive behavior with audio-visual dual-channel in panoramic digital museum. Inf. Manag. 60:103791. doi: 10.1016/j.im.2023.103791

[B24] Huangfu Q. Deng T. Guo Y. Li Y. Feng R. Wang Z. . (2026). Prior knowledge interacts with the effects of pre-questions and feedback types on learning from videos: eye-tracking and cognitive load evidence. Br. J. Educ. Technol. 57, 1115–1139. doi: 10.1111/bjet.70046

[B25] Iddamalgoda C. Ng K. H. Koleva B. Generative A. I. (2026). for supporting cultural learning and reflection: a study on technology user acceptance. Int. J. Hum.-Comput. Interact. 42, 197–210. doi: 10.1080/10447318.2025.2505777

[B26] Jegede O. J. Aikenhead G. S. (1999). Transcending cultural borders: implications for science teaching. Res. Sci. Technol. Educ. 17, 45–66. doi: 10.1080/0263514990170104

[B27] Kala N. Ayas A. (2023). Effect of instructional design based on cognitive load theory on students' performances and the indicators of element interactivity. J. Turk. Sci. Educ. 20, 468–489. doi: 10.36681/tused.2023.027

[B28] Lai S. Zhang Q. (2025). Exploring the determinants of users' intention to use augmented reality in craftsmanship-oriented intangible cultural heritage games. SAGE Open 15. doi: 10.1177/21582440251359021

[B29] Li J. Zhou L. Wei W. (2025). Analyzing factors influencing learning motivation in online virtual museums using the SOR model: a case study of the National Museum of Natural History. Information 16:573. doi: 10.3390/info16070573

[B30] Li Q. Chen Z. Wu T. Shen C. Yang Y. Zhao X. . (2026). Exploring the effects of narrative augmented reality in cultural heritage: a study on emotional arousal, intrinsic motivation, cognitive load, and learning. Int. J. Hum.-Comput. Interact. 42, 16–26. doi: 10.1080/10447318.2025.2504185

[B31] Li Q. Wang P. Liu Z. Zhang H. Song Y. Zhang Y. . (2024). Using scaffolding theory in serious games to enhance traditional Chinese murals culture learning. Comput. Animat. Virtual Worlds 35:e2213. doi: 10.1002/cav.2213

[B32] LyuC. TangS. LiS. (2026). AI interactivity and human-technology engagement: psychological mechanisms underlying learners' intention to use AI tools in language learning contexts. BMC Psychol. 14:432. doi: 10.1186/s40359-026-04229-741724987 PMC13037126

[B33] Machlanski D. Samothrakis S. Clarke P. S. (2024). Robustness of algorithms for causal structure learning to hyperparameter choice, causal learning and reasoning. PMLR 2024, 703–739.

[B34] Marangunić N. Granić A. (2015). Technology acceptance model: a literature review from 1986 to 2013. Universal Access Inf. Soc. 14, 81–95. doi: 10.1007/s10209-014-0348-1

[B35] Meng W. Dolah J. (2025). From virtual museum experience quality to offline visit intention: a cultural identity mediation model for sustainable heritage engagement. Sustainability 17:10664. doi: 10.3390/su172310664

[B36] Miao F. Holmes W. (2021). AI and Education: A Guidance for Policymakers. Paris: UNESCO Publishing.

[B37] Miao J. Bahauddin A. Feng J. (2024). Museum fatigue: spatial design narrative strategies of the Mawangdui Han Tomb. Buildings 14:3852. doi: 10.3390/buildings14123852

[B38] Mo J. Chen H. Ye C. Wang Z. Chen C. (2026). Exploring the drivers of users' adoption of museum digital humans. npj Heritage Sci. 14:43. doi: 10.1038/s40494-026-02313-0

[B39] Moghavvemi S. Jam F. A. (2025). Unraveling the influential factors driving persistent adoption of ChatGPT in learning environments. Educ. Inf. Technol. 30, 22443–22470. doi: 10.1007/s10639-025-13662-x

[B40] Pegolo G. (2023). Artificial intelligence for museums. A comparison within Italian chatbot applications (thesis). Università Ca' Foscari, Venice, Italy.

[B41] Pescapè A. (2024). Exploring the Current State and Future Potential of Generative Artificial Intelligence Using a Generative Artificial Intelligence, Mind, Body, and Digital Brains. Cham: Springer, 37–56. doi: 10.1007/978-3-031-58363-6_4

[B42] Qi J. Zhao H. Xiang R. Zhang Y. Zhang X. Wang C. . (2025). Understanding the interaction between generative AI and factual knowledge in supporting students' problem-solving. J. Res. Technol. Educ. 1–23. doi: 10.1080/15391523.2025.2601067

[B43] Ragin C. C. (2009). Redesigning Social Inquiry: Fuzzy Sets and Beyond. Chicago, IL: University of Chicago Press. doi: 10.7208/chicago/9780226702797.001.0001

[B44] RenJ. GuoJ. LiH. (2025). Linking digital competence, self-efficacy, and digital stress to perceived interactivity in AI-supported learning contexts. Sci. Rep. 15:33182. doi: 10.1038/s41598-025-18873-341006796 PMC12475259

[B45] Roca J. C. Chiu C-. M. Martínez F. J. (2006). Understanding e-learning continuance intention: an extension of the technology acceptance model. Int. J. Hum.-Comput. Stud. 64, 683–696. doi: 10.1016/j.ijhcs.2006.01.003

[B46] Rosé C. P. McLaughlin E. A. Liu R. Koedinger K. R. (2019). Explanatory learner models: why machine learning (alone) is not the answer. Br. J. Educ. Technol. 50, 2943–2958. doi: 10.1111/bjet.12858

[B47] Sáiz Manzanares M. C. Rodríguez Diez J. J. Marticorena Sánchez R. Zaparain Yanez M. J. Cerezo Menéndez R. (2020). Lifelong learning from sustainable education: an analysis with eye tracking and data mining techniques. Sustainability 12:1970. doi: 10.3390/su12051970

[B48] Sharma S. Khadka A. (2026). Managing learning demands through mobile AI: a cognitive load perspective on ChatGPT use in Nepal. Mobile Media Commun. doi: 10.1177/20501579251407518

[B49] Shephard K. (2008). Higher education for sustainability: seeking affective learning outcomes. Int. J. Sustainability Higher Educ. 9, 87–98. doi: 10.1108/14676370810842201

[B50] ShiM. DengL. ZhangM. LongY. (2025a). How telepresence and perceived enjoyment mediate the relationship between interaction quality and continuance intention: evidence from China Zisha-ware Digital Museum. PLoS ONE 20:e0317784. doi: 10.1371/journal.pone.031778439841733 PMC11753657

[B51] Shi M. Zhang M. Chen J. Zhu Y. (2025b). Perceived platform quality and user satisfaction in China Zisha-ware digital museum: mediating roles of confirmation and perceived usefulness. SAGE Open 15. doi: 10.1177/21582440251376490

[B52] Skulmowski A. Xu K. M. (2022). Understanding cognitive load in digital and online learning: a new perspective on extraneous cognitive load. Educ. Psychol. Rev. 34, 171–196. doi: 10.1007/s10648-021-09624-7

[B53] Susanto P. Mutalif Q. A. Setiana D. Besar N. (2025). From usefulness to motivation: a TAM-SDT perspective on AI adoption and problematic internet use in higher education. Front. Educ. 10:1699827. doi: 10.3389/feduc.2025.1699827

[B54] Sweller J. (2010). Element interactivity and intrinsic, extraneous, and germane cognitive load. Educ. Psychol. Rev. 22, 123–138. doi: 10.1007/s10648-010-9128-5

[B55] Tang W. Zhang X. Tian Y. (2023). Investigating lifelong learners' continuing learning intention moderated by affective support in online learning. Sustainability 15:1901. doi: 10.3390/su15031901

[B56] Wu X. Chen X. Zhao J. Xie Y. (2024). Influences of design and knowledge type of interactive virtual museums on learning outcomes: an eye-tracking evidence-based study. Educ. Inf. Technol. 29, 7223–7258. doi: 10.1007/s10639-023-12061-4

[B57] Xia B. Lei Y. Hu Y. Zhu X. Zhang J. (2026). Sustainable use intention of text-to-image generative AI in higher education: an S-O-R model with parallel trust and risk pathways. Sustainability 18:1657. doi: 10.3390/su18031657

[B58] Yan S. Eng L. G. Seong L. C. (2024). Influencing factors of continuous intention to use E-learning system of undergraduates in Guangxi, China: the mediating role of perceived ease of use and perceived usefulness. Sage Open 14, 1–21. doi: 10.1177/21582440241305231

[B59] Yilmaz K. T. Yilmaz Z. Arpacik Ö. (2025). Gamified mobile guidance for museum virtual tours: how task design shapes learning experience and cognitive load. J. Educ. Comput. Res. 63, 1430–1459. doi: 10.1177/07356331251347008

[B60] Zheng H. Qian Y. Wang Z. Wu Y. (2023). Research on the influence of E-learning quality on the intention to continue E-learning: evidence from SEM and fsQCA. Sustainability 15:5557. doi: 10.3390/su15065557

[B61] Zhou T. Ma X. (2025). Examining generative AI user continuance intention based on the SOR model. Aslib J. Inf. Manag. 78, 969–987. doi: 10.1108/AJIM-08-2024-0620

[B62] Zhou W. Xue Y. Wang S. Cang M. Qi K. Qiao Z. . (2025). Cognitive load-based multi-level annotation model for knowledge acquisition in heritage games. npj Heritage Sci. 13:559. doi: 10.1038/s40494-025-02133-8

[B63] Zhu L. Li H. Wang F-. K. He W. Tian Z. (2020). How online reviews affect purchase intention: a new model based on the stimulus-organism-response (S-O-R) framework. Aslib J. Inf. Manag. 72, 463–488. doi: 10.1108/AJIM-11-2019-0308

