# Supplementary material for: Cognitive load, prior knowledge, and sustained learning intention in a generative-AI-supported digital cultural learning context
Source: Front Psychol. 2026 Jul 3;17:1897481. doi: 10.3389/fpsyg.2026.1897481 (PMC13377363; doi:10.3389/fpsyg.2026.1897481)
Supplement: Supplementary file 1 [file Supplementary_file_1.docx]

Supplementary Material

Appendix A.

| **Layer** | **Construct** | **Theoretical basis** | **Measurement source(s)** | **Adaptation in this study** |
| --- | --- | --- | --- | --- |
| S | Information Quality (IQ) | D&M; S-O-R | [1-3] | Adapted to learners’ perceived quality of GenAI-provided traditional-culture learning information, specifically the accuracy, relevance, completeness, clarity, and understandability of AI-generated explanations, answers, and content outputs. |
| S | Perceived Ease of Use (PEOU) | TAM; S-O-R | [4-6] | Adapted to the perceived ease of prompt entry, function operation, output retrieval, and overall process navigation in GenAI-assisted traditional-culture learning. |
| S | Perceived Interactivity (PINT) | Interactivity / HCI | [7-9] | Adapted to learners’ perceived level of timely feedback, personalized response, process controllability, and sustained interaction support during GenAI-assisted traditional-culture learning. |
| O | Intrinsic Cognitive Load (ICL) | CLT | [10] | Adapted to the necessary cognitive processing burden caused by the complexity of traditional-culture content itself, including historical background, symbolic meaning, conceptual relations, and semantic abstraction. |
| O | Extraneous Cognitive Load (ECL) | CLT | [10, 11] | Adapted to the unnecessary cognitive burden arising from fragmented presentation, redundant feedback, interface switching, and complex interaction paths in GenAI-assisted traditional-culture learning. |
| R | Sustained learning intention (SLI) | Post-adoption learning; S-O-R | [6, 12, 13] | Adapted to learners’ intention to continue encountering, further understanding, and persistently engaging in traditional-culture learning after a GenAI-assisted learning experience. |
| Moderator | Prior Cultural Knowledge (PCK) | Schema theory; CLT | [14-16] | Adapted to learners’ prior knowledge reserve regarding relevant traditional-culture content, historical background, and core concepts before using GenAI for learning. |

Appendix B.

| **Variable** | **Full membership** | **Crossover point** | **Full non-membership** |
| --- | --- | --- | --- |
| IQ | 6.250 | 6.000 | 3.750 |
| PEOU | 6.250 | 6.000 | 3.750 |
| PINT | 4.000 | 2.000 | 1.750 |
| ICL | 4.750 | 2.000 | 1.750 |
| ECL | 5.500 | 2.250 | 1.750 |
| PCK | 6.250 | 6.000 | 4.250 |
| SLI | 6.250 | 6.000 | 3.750 |

Appendix C. Truth-table settings for fsQCA

| **Item** | **Setting in this study** |
| --- | --- |
| Software | fsQCA 3.0 |
| Outcome variable | Sustained learning intention (SLI) |
| Conditions | Information quality (IQ), perceived ease of use (PEOU), perceived interactivity (PINT), intrinsic cognitive load (ICL), extraneous cognitive load (ECL), and prior cultural knowledge (PCK) |
| Calibration method | Direct calibration |
| Full membership | 75th percentile |
| Crossover point | 50th percentile |
| Full non-membership | 25th percentile |
| Adjustment of crossover value | Values calibrated exactly at 0.500 were adjusted to 0.501 |
| Case-frequency threshold | 5 |
| Raw consistency threshold | 0.80 |
| PRI consistency threshold | 0.75 |
| Primary solution for interpretation | Intermediate solution |
| Contradictory configuration handling | Rows that did not meet the case-frequency, raw consistency, and PRI consistency thresholds were not included in the final sufficient-condition solutions |

Appendix D. Robustness checks for fsQCA

| **Analysis** | **Case-frequency threshold** | **Raw consistency threshold** | **PRI consistency threshold** | **Main result** |
| --- | --- | --- | --- | --- |
| Main analysis | 5 | 0.80 | 0.75 | Five configurations for high SLI and three configurations for low SLI were identified. |
| Robustness check | 6 | 0.85 | 0.75 | The resulting configurations remained substantively consistent with the original results. The new pathways were subsets of the original pathways, and no contradictory substantive conclusion emerged. |

Appendix E. Full-collinearity VIF results for common method bias assessment

| **Construct** | **Full-collinearity VIF** | |
| --- | --- | --- |
| Information quality (IQ) | 1.502 |  |
| Perceived ease of use (PEOU) | 1.531 |  |
| Perceived interactivity (PINT) | 1.442 |  |
| Intrinsic cognitive load (ICL) | 1.812 |  |
| Extraneous cognitive load (ECL) | 1.326 |  |
| Prior cultural knowledge (PCK) | 1.524 |  |
| Sustained learning intention (SLI) | 1.459 |  |

*Note.* Full-collinearity VIF values were examined as an additional diagnostic for common method bias. All values were below the strict threshold of 3.3, suggesting that common method bias was unlikely to severely distort the results.

Appendix F. Complete truth tables for fsQCA

Appendix F1. Truth table for high sustained learning intention

| **IQ** | **PEOU** | **PINT** | **ICL** | **ECL** | **PCK** | **Number of cases** | **High SLI** | **Raw consistency** | **PRI consistency** | **SYM consistency** |
| --- | --- | --- | --- | --- | --- | --- | --- | --- | --- | --- |
| 1 | 0 | 1 | 0 | 0 | 1 | 7 | 1 | 0.927 | 0.874 | 0.874 |
| 1 | 0 | 0 | 0 | 0 | 1 | 6 | 1 | 0.933 | 0.872 | 0.872 |
| 0 | 1 | 1 | 0 | 0 | 1 | 9 | 1 | 0.908 | 0.841 | 0.841 |
| 1 | 0 | 1 | 0 | 1 | 1 | 10 | 1 | 0.919 | 0.834 | 0.834 |
| 1 | 0 | 1 | 1 | 0 | 1 | 6 | 1 | 0.913 | 0.832 | 0.832 |
| 1 | 1 | 1 | 0 | 1 | 1 | 9 | 1 | 0.909 | 0.805 | 0.816 |
| 1 | 1 | 1 | 0 | 0 | 1 | 32 | 1 | 0.874 | 0.791 | 0.804 |
| 1 | 0 | 0 | 1 | 0 | 1 | 7 | 1 | 0.887 | 0.783 | 0.787 |
| 0 | 0 | 1 | 1 | 0 | 1 | 20 | 1 | 0.873 | 0.783 | 0.786 |
| 1 | 1 | 0 | 0 | 1 | 1 | 12 | 1 | 0.887 | 0.764 | 0.826 |
| 0 | 0 | 1 | 0 | 0 | 1 | 16 | 0 | 0.844 | 0.748 | 0.788 |
| 1 | 1 | 0 | 0 | 0 | 0 | 5 | 0 | 0.877 | 0.747 | 0.747 |
| 0 | 1 | 0 | 0 | 0 | 1 | 11 | 0 | 0.846 | 0.744 | 0.744 |
| 1 | 1 | 0 | 0 | 0 | 1 | 15 | 0 | 0.859 | 0.737 | 0.753 |
| 1 | 1 | 1 | 0 | 0 | 0 | 7 | 0 | 0.856 | 0.732 | 0.732 |
| 1 | 1 | 0 | 1 | 0 | 1 | 20 | 0 | 0.850 | 0.729 | 0.738 |
| 1 | 1 | 1 | 1 | 0 | 1 | 24 | 0 | 0.843 | 0.726 | 0.736 |
| 0 | 1 | 1 | 0 | 1 | 1 | 8 | 0 | 0.896 | 0.721 | 0.721 |
| 0 | 1 | 0 | 1 | 0 | 1 | 11 | 0 | 0.819 | 0.699 | 0.699 |
| 0 | 1 | 1 | 1 | 0 | 1 | 8 | 0 | 0.837 | 0.670 | 0.670 |
| 0 | 0 | 1 | 0 | 1 | 1 | 22 | 0 | 0.805 | 0.659 | 0.759 |
| 1 | 1 | 0 | 1 | 0 | 0 | 9 | 0 | 0.777 | 0.611 | 0.611 |
| 1 | 0 | 1 | 1 | 1 | 1 | 9 | 0 | 0.770 | 0.582 | 0.614 |
| 0 | 0 | 1 | 1 | 1 | 1 | 26 | 0 | 0.727 | 0.542 | 0.549 |
| 1 | 1 | 1 | 1 | 1 | 1 | 12 | 0 | 0.767 | 0.527 | 0.527 |
| 1 | 1 | 0 | 1 | 1 | 1 | 14 | 0 | 0.715 | 0.504 | 0.504 |
| 0 | 0 | 1 | 1 | 0 | 0 | 9 | 0 | 0.696 | 0.491 | 0.492 |
| 0 | 1 | 1 | 1 | 0 | 0 | 8 | 0 | 0.761 | 0.482 | 0.482 |
| 0 | 1 | 0 | 1 | 0 | 0 | 5 | 0 | 0.680 | 0.482 | 0.482 |
| 0 | 1 | 1 | 1 | 1 | 1 | 6 | 0 | 0.721 | 0.403 | 0.426 |
| 1 | 1 | 0 | 1 | 1 | 0 | 27 | 0 | 0.466 | 0.259 | 0.259 |
| 1 | 0 | 0 | 1 | 1 | 0 | 10 | 0 | 0.467 | 0.223 | 0.225 |
| 1 | 1 | 1 | 1 | 1 | 0 | 24 | 0 | 0.503 | 0.204 | 0.205 |
| 1 | 0 | 1 | 1 | 1 | 0 | 13 | 0 | 0.456 | 0.187 | 0.187 |
| 0 | 1 | 0 | 1 | 1 | 0 | 9 | 0 | 0.439 | 0.176 | 0.177 |
| 0 | 1 | 1 | 1 | 1 | 0 | 8 | 0 | 0.470 | 0.173 | 0.173 |
| 0 | 0 | 1 | 1 | 1 | 0 | 57 | 0 | 0.297 | 0.117 | 0.121 |

Appendix F2. Truth table for low sustained learning intention

| **IQ** | **PEOU** | **PINT** | **ICL** | **ECL** | **PCK** | **Number of cases** | **Low SLI** | **Raw consistency** | **PRI consistency** | **SYM consistency** |
| --- | --- | --- | --- | --- | --- | --- | --- | --- | --- | --- |
| 0 | 0 | 1 | 1 | 1 | 0 | 57 | 1 | 0.876 | 0.844 | 0.879 |
| 0 | 1 | 1 | 1 | 1 | 0 | 8 | 1 | 0.889 | 0.827 | 0.827 |
| 0 | 1 | 0 | 1 | 1 | 0 | 9 | 1 | 0.877 | 0.819 | 0.823 |
| 1 | 0 | 1 | 1 | 1 | 0 | 13 | 1 | 0.872 | 0.809 | 0.813 |
| 1 | 1 | 1 | 1 | 1 | 0 | 24 | 1 | 0.870 | 0.791 | 0.795 |
| 1 | 0 | 0 | 1 | 1 | 0 | 10 | 1 | 0.843 | 0.771 | 0.775 |
| 1 | 1 | 0 | 1 | 1 | 0 | 27 | 0 | 0.813 | 0.741 | 0.741 |
| 0 | 1 | 1 | 1 | 1 | 1 | 6 | 0 | 0.786 | 0.543 | 0.574 |
| 0 | 1 | 0 | 1 | 0 | 0 | 5 | 0 | 0.702 | 0.518 | 0.518 |
| 0 | 1 | 1 | 1 | 0 | 0 | 8 | 0 | 0.777 | 0.518 | 0.518 |
| 0 | 0 | 1 | 1 | 0 | 0 | 9 | 0 | 0.705 | 0.506 | 0.508 |
| 1 | 1 | 0 | 1 | 1 | 1 | 14 | 0 | 0.710 | 0.496 | 0.496 |
| 1 | 1 | 1 | 1 | 1 | 1 | 12 | 0 | 0.741 | 0.473 | 0.473 |
| 0 | 0 | 1 | 1 | 1 | 1 | 26 | 0 | 0.670 | 0.445 | 0.451 |
| 1 | 1 | 0 | 1 | 0 | 0 | 9 | 0 | 0.650 | 0.389 | 0.389 |
| 1 | 0 | 1 | 1 | 1 | 1 | 9 | 0 | 0.651 | 0.365 | 0.386 |
| 0 | 1 | 1 | 1 | 0 | 1 | 8 | 0 | 0.669 | 0.330 | 0.330 |
| 0 | 1 | 0 | 1 | 0 | 1 | 11 | 0 | 0.578 | 0.301 | 0.301 |
| 0 | 1 | 1 | 0 | 1 | 1 | 8 | 0 | 0.730 | 0.279 | 0.279 |
| 1 | 1 | 1 | 0 | 0 | 0 | 7 | 0 | 0.605 | 0.268 | 0.268 |
| 1 | 1 | 1 | 1 | 0 | 1 | 24 | 0 | 0.575 | 0.260 | 0.264 |
| 1 | 1 | 0 | 1 | 0 | 1 | 20 | 0 | 0.589 | 0.259 | 0.262 |
| 0 | 1 | 0 | 0 | 0 | 1 | 11 | 0 | 0.551 | 0.256 | 0.256 |
| 1 | 1 | 0 | 0 | 0 | 0 | 5 | 0 | 0.636 | 0.253 | 0.253 |
| 1 | 1 | 0 | 0 | 0 | 1 | 15 | 0 | 0.595 | 0.242 | 0.247 |
| 0 | 0 | 1 | 1 | 0 | 1 | 20 | 0 | 0.539 | 0.213 | 0.214 |
| 1 | 0 | 0 | 1 | 0 | 1 | 7 | 0 | 0.590 | 0.212 | 0.213 |
| 0 | 0 | 1 | 0 | 1 | 1 | 22 | 0 | 0.548 | 0.209 | 0.241 |
| 0 | 0 | 1 | 0 | 0 | 1 | 16 | 0 | 0.504 | 0.201 | 0.212 |
| 1 | 1 | 1 | 0 | 0 | 1 | 32 | 0 | 0.512 | 0.193 | 0.196 |
| 1 | 1 | 1 | 0 | 1 | 1 | 9 | 0 | 0.620 | 0.181 | 0.184 |
| 1 | 0 | 1 | 1 | 0 | 1 | 6 | 0 | 0.566 | 0.168 | 0.168 |
| 1 | 0 | 1 | 0 | 1 | 1 | 10 | 0 | 0.594 | 0.166 | 0.166 |
| 1 | 1 | 0 | 0 | 1 | 1 | 12 | 0 | 0.599 | 0.161 | 0.174 |
| 0 | 1 | 1 | 0 | 0 | 1 | 9 | 0 | 0.515 | 0.159 | 0.159 |
| 1 | 0 | 0 | 0 | 0 | 1 | 6 | 0 | 0.543 | 0.128 | 0.128 |
| 1 | 0 | 1 | 0 | 0 | 1 | 7 | 0 | 0.490 | 0.126 | 0.126 |

*Note.* In the truth tables, 1 indicates the presence of a calibrated condition and 0 indicates its absence. Rows that did not meet the case-frequency, raw consistency, and PRI consistency thresholds were not included in the final sufficient-condition solutions.

Appendix G. Additional fsQCA solution outputs

Appendix G1. Complex solution for high sustained learning intention

| **Configuration** | **Raw coverage** | **Unique coverage** | **Consistency** |
| --- | --- | --- | --- |
| IQ*~PEOU*~ECL*PCK | 0.181 | 0.055 | 0.912 |
| IQ**PINT*~ICL*PCK | 0.215 | 0.024 | 0.870 |
| PEOU**PINT*~ICL*~ECL**PCK | 0.170 | 0.023 | 0.881 |
| ~PEOU**PINT**ICL*~ECL**PCK | 0.105 | 0.037 | 0.876 |
| IQ**PEOU*~ICL**ECL**PCK | 0.138 | 0.049 | 0.882 |
| Overall solution consistency | 0.868 |  |  |
| Overall solution coverage | 0.408 |  |  |

Appendix G2. Parsimonious solution for high sustained learning intention

| **Configuration** | **Raw coverage** | **Unique coverage** | **Consistency** |
| --- | --- | --- | --- |
| IQ*~ICL**ECL | 0.195 | 0.073 | 0.808 |
| ~PEOU**ICL*~ECL*PCK | 0.150 | 0.050 | 0.873 |
| PEOU**PINT*~ICL*~ECL**PCK | 0.170 | 0.067 | 0.881 |
| IQ*~PEOU*~ECL | 0.222 | 0.014 | 0.870 |
| IQ*~PEOU*~ICL | 0.236 | 0.003 | 0.868 |
| Overall solution consistency | 0.832 |  |  |
| Overall solution coverage | 0.480 |  |  |

Appendix G3. Complex solution for low sustained learning intention

| **Configuration** | **Raw coverage** | **Unique coverage** | **Consistency** |
| --- | --- | --- | --- |
| PINT**ICL**ECL*~PCK | 0.278 | 0.157 | 0.873 |
| IQ*~PEOU**ICL**ECL*~PCK | 0.127 | 0.030 | 0.845 |
| ~IQ**PEOU**ICL**ECL*~PCK | 0.119 | 0.028 | 0.894 |
| Overall solution consistency | 0.866 |  |  |
| Overall solution coverage | 0.352 |  |  |

Appendix G4. Parsimonious solution for low sustained learning intention

| **Configuration** | **Raw coverage** | **Unique coverage** | **Consistency** |
| --- | --- | --- | --- |
| ~IQ**ECL*~PCK | 0.298 | 0.030 | 0.848 |
| PINT**ECL*~PCK | 0.303 | 0.039 | 0.838 |
| ~PEOU*~PINT*~PCK | 0.182 | 0.009 | 0.703 |
| IQ*~PEOU*~PCK | 0.184 | 0.008 | 0.674 |
| ~PEOU**ECL*~PCK | 0.313 | 0.006 | 0.828 |
| ~PEOU*~PINT**ECL | 0.194 | 0.050 | 0.735 |
| Overall solution consistency | 0.736 |  |  |
| Overall solution coverage | 0.515 |  |  |

*Note.* The tilde symbol (~) indicates the absence of a condition. The intermediate solution is reported in the main text because it was used as the primary interpretive basis.

Appendix H. Detailed robustness-check solution

To assess the robustness of the fsQCA findings, the raw consistency threshold was increased from 0.80 to 0.85, and the case-frequency threshold was increased from 5 to 6. Under the stricter threshold settings, the high-SLI configurations remained substantively consistent with the original solution. For low sustained learning intention, the solution converged from three pathways to two pathways, as shown below.

| **Condition** | **L1** | **L2** |
| --- | --- | --- |
| IQ |  | ⨂ |
| PEOU |  | ● |
| PINT | ● |  |
| ICL | ● | ● |
| ECL | ● | ● |
| PCK | ⨂ | ⨂ |
| Consistency | 0.873 | 0.894 |
| Raw coverage | 0.278 | 0.119 |
| Unique coverage | 0.203 | 0.044 |
| Overall solution consistency | 0.877 |  |
| Overall solution coverage | 0.322 |  |

*Note.* ● indicates the presence of a core condition; ⨂ indicates the absence of a core condition; blank cells indicate that the condition may be either present or absent. The robustness-check solution remained substantively consistent with the original findings, and no contradictory substantive conclusion emerged.

[1] W. H. DeLone and E. R. McLean, "The DeLone and McLean model of information systems success: a ten-year update," *Journal of management information systems,* vol. 19, no. 4, pp. 9-30, 2003.

[2] M. Shi, M. Zhang, J. Chen, and Y. Zhu, "Perceived Platform Quality and User Satisfaction in China Zisha-ware Digital Museum: Mediating Roles of Confirmation and Perceived Usefulness," *SAGE Open,* vol. 15, no. 3, p. 21582440251376490, 2025.

[3] B. Xia, Y. Lei, Y. Hu, X. Zhu, and J. Zhang, "Sustainable Use Intention of Text-to-Image Generative AI in Higher Education: An S–O–R Model with Parallel Trust and Risk Pathways," *Sustainability,* vol. 18, no. 3, p. 1657, 2026.

[4] F. D. Davis, "Perceived usefulness, perceived ease of use, and user acceptance of information technology," *MIS quarterly,* vol. 13, no. 3, pp. 319-340, 1989.

[5] S. Yan, L. G. Eng, and L. C. Seong, "Influencing factors of continuous intention to use E-learning system of undergraduates in Guangxi, China: The mediating role of perceived ease of use and perceived usefulness," *Sage Open,* vol. 14, no. 4, p. 21582440241305231, 2024.

[6] J. Guo, H. Ren, and Y. Qi, "Exploring factors influencing students’ sustainable learning intention in the application of augmented reality in education: a case study in radio and television scripting and directing," *Interactive Learning Environments,* vol. 33, no. 10, pp. 5726-5746, 2025.

[7] Y.-M. Cheng, "Roles of interactivity and usage experience in e-learning acceptance: a longitudinal study," *International Journal of Web Information Systems,* vol. 10, no. 1, pp. 2-23, 2014.

[8] C. Lyu, S. Tang, and S. Li, "AI interactivity and human-technology engagement: psychological mechanisms underlying learners’ intention to use AI tools in language learning contexts," *BMC psychology,* 2026.

[9] W. N. Alwakid, N. A. Dahri, M. Humayun, and G. N. Alwakid, "Integrating AI chatbots for enhancing academic support in business education: A SEM-Based study toward sustainable learning," *The International Journal of Management Education,* vol. 23, no. 3, p. 101252, 2025.

[10] J. Leppink, F. Paas, C. P. Van der Vleuten, T. Van Gog, and J. J. Van Merriënboer, "Development of an instrument for measuring different types of cognitive load," *Behavior research methods,* vol. 45, no. 4, pp. 1058-1072, 2013.

[11] A. Skulmowski and K. M. Xu, "Understanding cognitive load in digital and online learning: A new perspective on extraneous cognitive load," *Educational psychology review,* vol. 34, no. 1, pp. 171-196, 2022.

[12] S. Fu, H. Gu, and B. Yang, "The affordances of AI‐enabled automatic scoring applications on learners’ continuous learning intention: An empirical study in China," *British Journal of Educational Technology,* vol. 51, no. 5, pp. 1674-1692, 2020.

[13] W. Tang, X. Zhang, and Y. Tian, "Investigating lifelong learners’ continuing learning intention moderated by affective support in online learning," *Sustainability,* vol. 15, no. 3, p. 1901, 2023.

[14] Z. Cai, C. Liu, Y. Yang, and B. Li, "The impact of learning supports in digital game-based learning on learners with different levels of prior knowledge," *The Internet and Higher Education,* p. 101044, 2025.

[15] Q. Huangfu, T. Deng, Y. Guo, Y. Li, R. Feng, and Z. Wang, "Prior knowledge interacts with the effects of pre‐questions and feedback types on learning from videos: Eye‐tracking and cognitive load evidence," *British Journal of Educational Technology,* 2026.

[16] A. Gorbunova, A. Kapuza, O. Chen, and J. Costley, "Rethinking pre-training: cognitive load implications for learners with varying prior knowledge," *Frontiers in Psychology,* vol. 16, p. 1628047, 2025.
